# Carbon catabolite repression involves physical interaction of the transcription factor CRE1/CreA and the Tup1–Cyc8 complex in *Penicillium oxalicum* and *Trichoderma reesei*

**DOI:** 10.1186/s13068-021-02092-9

**Published:** 2021-12-24

**Authors:** Yueyan Hu, Mengxue Li, Zhongjiao Liu, Xin Song, Yinbo Qu, Yuqi Qin

**Affiliations:** 1 National Glycoengineering Research Center, State Key Laboratory of Microbial Technology, Shandong University, No. 72 Binhai Road, Qingdao, 266237 China; 2 Shandong Key Laboratory of Carbohydrate Chemistry and Glycobiology, Shandong University, No. 72 Binhai Road, Qingdao, 266237 China; 3 NMPA Key Laboratory for Quality Research and Evaluation of Carbohydrate-Based Medicine, Shandong University, No. 72 Binhai Road, Qingdao, 266237 China

**Keywords:** CCR, Cellulases, CRE1, *Penicillium*, Transcription factor, *Trichoderma*

## Abstract

**Background:**

Cellulolytic enzyme production in filamentous fungi requires a release from carbon catabolite repression (CCR). The protein CRE1/CreA (CRE = catabolite responsive element) is a key transcription factor (TF) that is involved in CCR and represses cellulolytic gene expression. CRE1/CreA represents the functional equivalent of Mig1p, an important *Saccharomyces cerevisiae* TF in CCR that exerts its repressive effect by recruiting a corepressor complex Tup1p–Cyc8p. Although it is known from *S. cerevisiae* that CRE1/CreA might repress gene expression via interacting with the corepressor complex Tup1–Cyc8, this mechanism is unconfirmed in other filamentous fungi, since the physical interaction has not yet been verified in these organisms. The precise mechanism on how CRE1/CreA achieves transcriptional repression after DNA binding remains unknown.

**Results:**

The results from tandem affinity purification and bimolecular fluorescence complementation revealed a direct physical interaction between the TF CRE1/CreA and the complex Tup1–Cyc8 in the nucleus of cellulolytic fungus *Trichoderma reesei* and *Penicillium oxalicum*. Both fungi have the ability to secrete a complex arsenal of enzymes to synergistically degrade lignocellulosic materials. In *P*. *oxalicum*, the protein PoCyc8, a subunit of complex Tup1–Cyc8, interacts directly with TF PoCreA and histone H3 lysine 36 (H3K36) methyltransferase PoSet2 in the nucleus. The di-methylation level of H3K36 in the promoter of prominent cellulolytic genes (cellobiohydrolase-encoding gene Po*cbh1*/*cel7A* and endoglucanase-encoding gene Po*egl1*/*cel7B*) is positively correlated with the expression levels of TF PoCreA. Since the methylation of H3K36 was also demonstrated to be a repression marker of cellulolytic gene expression, it appears feasible that the cellulolytic genes are repressed via PoCreA-Tup1–Cyc8-Set2-mediated transcriptional repression.

**Conclusion:**

This study verifies the long-standing conjecture that the TF CRE1/CreA represses gene expression by interacting with the corepressor complex Tup1–Cyc8 in filamentous fungi. A reasonable explanation is proposed that PoCreA represses gene expression by recruiting complex PoTup1–Cyc8. Histone methyltransferase Set2, which methylates H3K36, is also involved in the regulatory network by interacting with PoCyc8. The findings contribute to the understanding of CCR mechanism in filamentous fungi and could aid in biotechnologically relevant enzyme production.

**Supplementary Information:**

The online version contains supplementary material available at 10.1186/s13068-021-02092-9.

## Introduction

Lignocellulosic biomass composed of polysaccharides (cellulose and hemicellulose) and an aromatic polymer (lignin) is the most abundant and highly renewable natural biological resource [1]. Many saprophytic fungi secrete different types of cellulolytic enzymes that degrade cellulose and hemicelluloses to a mixture of sugars (C-5 and C-6), which are then assimilated and metabolized by different microorganisms to participate in the global carbon cycle [2]. These sugars can also be fermented by industrial microorganisms to produce various chemicals, such as alcohols and organic acids [3, 4].

Cellulolytic enzyme production in filamentous fungi is tightly controlled at the transcription level. Cellulolytic gene expression is often repressed in the presence of preferentially utilized sugars (frequently glucose), a phenomenon known as carbon catabolite repression (CCR) [5]. In cellulolytic fungi, e.g., *Trichoderma*, *Aspergillus*, *Neurospora*, and *Penicillium*, CCR is mediated mainly by the transcription factor (TF) CRE1/CreA (CRE = catabolite responsive element), a C2H2 zinc finger protein that binds to the promoters of various genes repressed by glucose or xylose [6–8]. In *Aspergillus nidulans* and *Trichoderma reesei*, CRE1/CreA directly binds to 5ʹ-SYGGRG-3ʹ motif in the proximal promoter region and inhibits the expression of xylanase-encoding genes such as *xlnA*, *xlnB*, and *xlnD* [9] and cellulase-encoding genes such as *cbh1* (*cel7A*), *cbh2* (*cel6A*), and *eg2* (*cel5A*) [5, 10, 11]. Alterations in the subcellular localization of CRE1/CreA mediated by glucose concentration and posttranslational modification (specifically phosphorylation) are crucial for its regulation [12–14].

Cellulolytic gene induction requires a release from CCR. Therefore*,* the deletion, truncation, or multisite-directed mutagenesis of gene *cre1*/*creA* can alleviate CCR and thus improve the expression level of prominent cellulolytic genes in various carbon sources, such as glucose, lactose, sophorose, cellulose, or a mixture of plant polymers [5, 15–18]. For example, either the deletion or truncation of *cre1* in *T. reesei* wild-type strain QM6a leads to de-repressed production of cellulase and hemicellulase, when the mutants are cultivated in glucose-containing media [19]. The hypercellulolytic *T. reesei* strain Rut-C30, which can produce cellulase and hemicellulase in a medium containing glucose, has a truncated version of TrCRE1 [20]. Another hyperproducer of cellulolytic enzyme, *Penicillium oxalicum* JU-A10-T, has a frameshift mutation at the C-terminus of PoCreA, which plays a negative role on cellulolytic gene expression under repressed (glucose) or induced (cellulose) condition [8]. In addition, CRE1/CreA is crucial in many other biological processes, including asexual development, secondary metabolite production, glycogen metabolism, fungal virulence, and circadian rhythms in diverse fungi [21–24].

The regulating function of CRE1/CreA for the above biological processes ultimately originates from its controlling (specifically repression) roles for gene expression. However, the precise mechanism of transcriptional repression by CRE1/CreA after DNA binding remains unknown. The amino acid sequence of CRE1/CreA zinc finger region is similar to that of budding yeast Mig1p, an important TF in CCR [25]. In *Saccharomyces cerevisiae*, Mig1p exerts its repressive effect by recruiting corepressor complex Tup1p–Cyc8p (Ssn6) [26]. In filamentous fungi, Tup1p and Cyc8p have conserved homologous proteins, such as RcoA and SsnF in *A. nidulans*, RCO-1 and RCM-1 in *Neurospora crassa*, and TrTUP1 and TrCYC8 in *T. reesei* [21, 27–30]. Although it is known from *S. cerevisiae* that CRE1/CreA might repress gene expression via interacting with the corepressor complex Tup1–Cyc8, this mechanism is unconfirmed in other filamentous fungi, since the physical interaction between CRE1/CreA and the complex has not yet been verified in these organisms. *N. crassa* CRE-1, RCO-1, and RCM-1 proteins are involved in fungal development, glycogen accumulation, and phosphorylation-regulated glycogen synthase activity. However, whether *N. crassa* CRE-1 recruits the complex RCO-1/RCM-1 has not been proven [21, 29]. *T. reesei* TrTUP1 or TrCYC8 knockdown does not result in carbon catabolite de-repression [30]. García et al. showed that the absence of *rcoA* (the homologue of yeast Tup1p) does not cause carbon catabolite de-repression in *A. nidulans* [27]. The cohesive picture of gene repression mediated by CRE1/CreA in filamentous fungi has never been explored.

In this study, tandem affinity purification (TAP) and bimolecular fluorescence complementation (BiFC) were used to verify the direct physical interaction between the TF CRE1/CreA and the complex Tup1–Cyc8 in *T*. *reesei* and *P*. *oxalicum*. A reasonable explanation on how PoCreA represses gene expression by recruiting Tup1–Cyc8 was also presented.

## Results

### TrTUP1 and TrCYC8 are protein–protein interaction partners of *T. reesei* TrCRE1

Eukaryotic TFs regulate transcription by recruiting cofactors that control the specific phases of transcription. TAP is a purification technique for protein–protein interaction analysis that incorporates an epitope tag (TAP tag) onto the protein of interest and performs a two-step affinity purification protocol to isolate TAP-tagged proteins and associated proteins. This two-step purification process reduces the amount of non-specific binding proteins. TAP coupled with mass-spectrometry (TAP-MS) for CRE1/CreA was performed in *T. reesei* and *P. oxalicum* to identify the putative cofactors of CRE1/CreA. First, TAP-MS for *T. reesei* TrCRE1-labeled strain (TrCRE1-FLAG-HA) was conducted to identify the protein–protein interaction collaborator of TrCRE1. The gene encoding for TrCRE1 was C-terminally fused with the FLAG (DYKDDDDK) and HA (YPYDVPDYA) tags (theoretical molecular weight (MW): 4.28 kDa) and then transformed into the parent strain *T. reesei* QP4 [31] to substitute the native Tr*cre1* gene. The corresponding strain was named TrCRE1-TAP. The strains are listed in Additional file 1: Table S1.

No significant difference in mycelia growth and conidia production was observed between the TrCRE1-TAP and the parent strain. In particular, their cellulolytic genes had a similar expression pattern (Additional file 2: Fig. S1A, B), indicating the lack of biological interference from the insertion of FLAG and HA tags. For TAP-MS experiments, TAP eluents from the parent strain *T. reesei* QP4 were used as the control. The final TAP eluents from the respective strains were divided into three parts for Western blot analysis, SDS-PAGE with silver staining, and LC–MS/MS to identify the bait and interacting proteins.

**Fig. 1:**
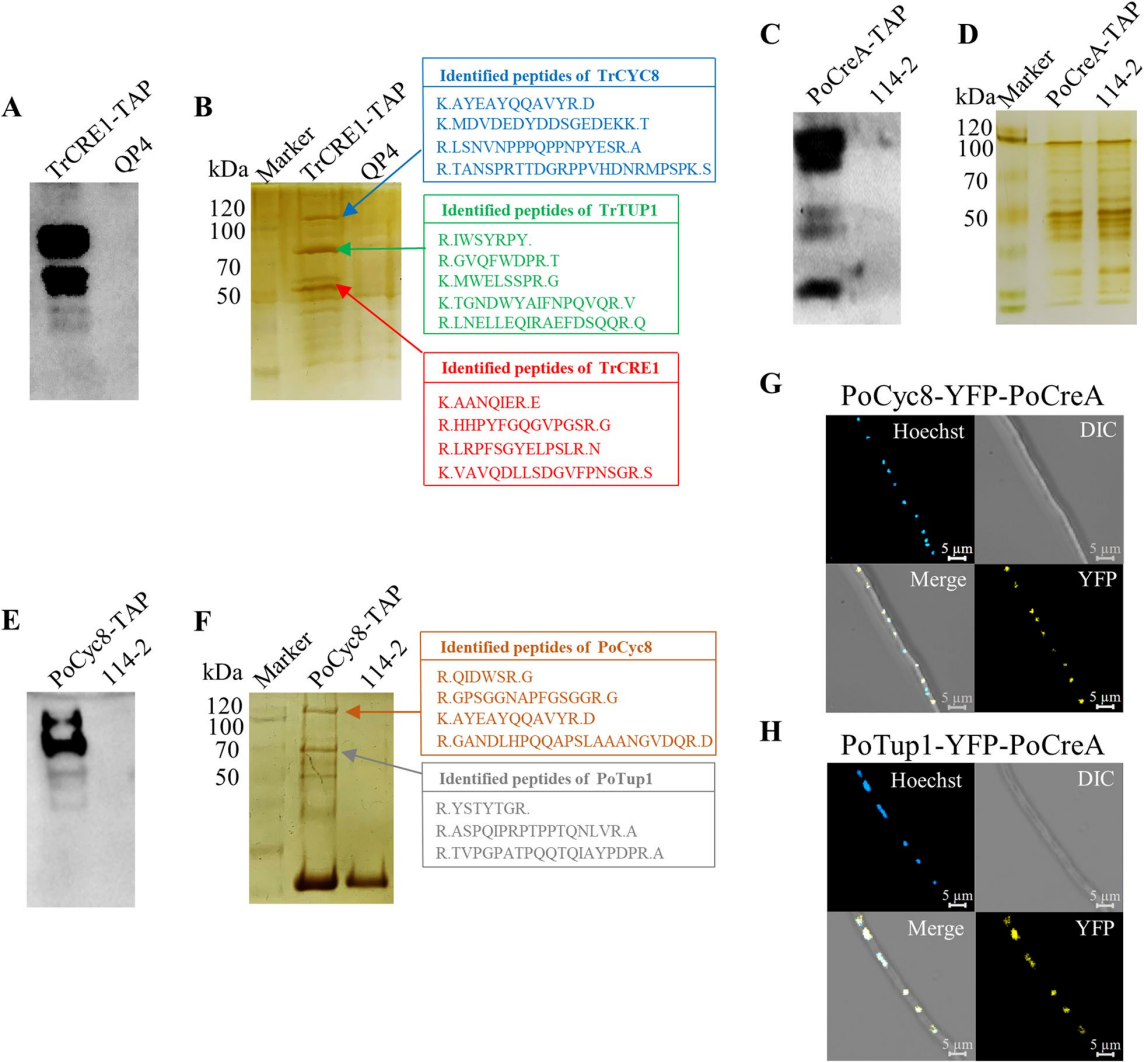
The results of TAP-MS and BiFC. Western blot (**A**) and SDS-PAGE with silver staining (**B**) of *T. reesei* QP4 (control) and TrCRE1-TAP strains. The green arrow, blue arrow, and red arrow represent proteins TrCYC8, TrTUP1, and TrCRE1, respectively. Western blot (**C**) and SDS-PAGE with silver staining (**D**) of *P. oxalicum* 114-2 (control) and PoCreA-TAP strains. Western blot (**E**) and SDS-PAGE with silver staining (**F**) of *P. oxalicum* 114-2 (control) and PoCyc8-TAP strains. The orange arrow and gray arrow represent proteins PoCyc8 and PoTup1, respectively. Western blot was performed using the ANTI-HA antibody (ABclonal, China). **G** Microscopy of PoCyc8-YFP-PoCreA BiFC strain; **H** Microscopy of PoTup1-YFP-PoCreA BiFC strain. Each image includes four parts. The upper left, blue particles indicate nucleus stained with Hoechst 33342. The upper right, normal white light. The bottom right, yellow fluorescent particles indicate interactions between two target proteins. The bottom left, indicating the merge of yellow fluorescence and blue nucleus

Western blot analysis indicated the existence of TrCRE1 bait (Fig. 1A). Several specific bands were found between the TrCRE1-TAP and its parent strain QP4 from the gel of SDS-PAGE with subsequent silver staining (Fig. 1B)*.* The bands were cut from the gel and identified by LC/MS–MS as TrCRE1 (Fig. 1B, red arrow, approximately 50 kDa, theoretical MW: 43.62 kDa), TrTUP1 (Fig. 1B, green arrow, approximately 70 kDa; theoretical MW: 66.00 kDa), and TrCYC8 (Fig. 1B, blue arrow, approximately 115 kDa, theoretical MW: 82.06 kDa). The proteins in the final eluent were identified by LC–MS/MS, and the TAP eluents from the parent strain *T. reesei* QP4 were used as the control. The proteins in all three TrCRE1-TAP samples but not in any of the controls were considered putative interacting proteins.

In addition to TrCRE1 itself as the bait, 37 protein targets of putative interactions with TrCRE1 were captured (Additional file 3: Spreadsheet S1). The top 10 proteins with the highest exponentially modified protein abundance index (emPAI) [32] are listed in Table 1. Among the top 10 proteins, TrCRE1 is the top one protein according to emPAI, TrTUP1 (Trire2_121940, the homologue of *S. cerevisiae* Tup1p) and TrCYC8 (Trire2_102616, the homologue of *S. cerevisiae* Cyc8p) are listed in the 3rd and 4th positions, respectively. Except for the complex, other putative proteins were listed in Table 1. These homologues of putative interacting proteins in *S. cerevisiae* are not completely localized in the nucleus. This finding is reasonable because CRE1/CreA is distributed in the nucleus and cytoplasm, even under repressed (glucose) condition [14, 33].

**Table 1:** The top 10 proteins interacting with TrCRE1 identified through TAP-MS

Rank	Protein ID	emPAI^a^	*T. reesei* QM6a	*S.* *cerevisiae* S288C				Predicted function
			Protein	Homologue	Identity %	*E* value	Location^b^	
1st	Trire2_120117	6.58 × 10^25^	TrCRE1	Mig1	72	7e−30	Nucleus and cytoplasm	Sequence-specific DNA binding transcription factor involved in the regulation of transcription by RNA polymerase II in response to glucose and starvation
2nd	Trire2_120053	1.09 × 10^5^	TrHSP70	Ssc1	74	0.0	Mitochondrion	Hsp70 family ATPase, involving in protein importing and folding
3rd	Trire2_121940	1.29 × 10^3^	TrTUP1	Tup1	49	2e−128	Nucleus	General repressor of transcription, forms complex with Cyc8p
4th	Trire2_102616	3.82 × 10^2^	TrCYC8	Cyc8	55	1e−136	Nucleus	General repressor of transcription, forms complex with Tup1p
5th	Trire2_122572	1.18 × 10^2^	–	Ssb2	73	0.0	Cytoplasm and membrane	Cytoplasmic ATPase that is a ribosome-associated molecular chaperone
6th	Trire2_122920	6.31	TrBIP1	Kar2	76	0.0	Endoplasmic reticulum	ATPase involved in protein import into the endoplasmic reticulum
7th	Trire2_121906	4.01	TrRPS14	Rps14A	84	6e−63	Nucleus and cytoplasm	mRNA-binding constituent of the cytosolic small ribosomal subunit; involved in maturation of the small subunit rRNA and assembly of the small ribosomal subunit
8th	Trire2_119855	3.96	TrRPS3	Rps3	75	3e−124	Cytoplasm	40 s ribosomal protein
9th	Trire2_102864	3.64	–	–	–	–	–	Uncharacterized protein
10th	Trire2_123753	2.98	TrRPS31	Rps31	85	8e−67	Nucleus and cytoplasm	Fusion protein that is cleaved to yield ubiquitin and a subunit of the cytosolic small ribosomal subunit; involved in maturation of the small subunit rRNA, assembly of the small ribosomal subunit, and translation

–: No significant similarity found

^a^emPAI is the Exponentially Modified Protein Abundance Index of three samples. Every Peptide count of each sample is listed in Additional file 3: Spreadsheet S1

^b^Data from Saccharomyces Genome Database (www.yeastgenome.org)

### PoTup1 and PoCyc8 are also protein–protein interaction partners of *P. oxalicum* PoCreA

*P. oxalicum* PoCreA-TAP strain was constructed using the same method for *T. reesei*, and the strains are listed in Additional file 1: Table S1. No significant difference in mycelia growth and conidia production was observed between the PoCreA-TAP and the parent strain. In particular, their cellulolytic genes had a similar expression pattern (Additional file 2: Fig. S1C, D), indicating the lack of biological interference from the insertion of FLAG and HA tags. Western blot analysis indicated the existence of PoCreA bait (approximately 50 kDa; theoretical MW: 44.95 kDa) (Fig. 1C), although no specific band was observed between the samples of PoCreA-TAP and parent strain 114-2 from the gel of SDS-PAGE with subsequent silver staining (Fig. 1D). The proteins in the final eluent were identified by LC–MS/MS, and the TAP eluents from the parent strain *P. oxalicum* 114-2 were used as the control. The proteins in all three PoCreA-TAP samples but not in any of the controls were considered putative interacting proteins.

In addition to PoCreA itself, 21 protein targets of putative interactions with PoCreA were captured (Additional file 3: Spreadsheet S1). The top 10 proteins with the highest emPAI are listed in Table 2. Among the top 10 proteins, the bait PoCreA showed the highest emPAI followed by PoTup1 (PDE_01024, the homologue of *S. cerevisiae* Tup1p) and PoCyc8 (PDE_03177, the homologue of *S. cerevisiae* Cyc8p) (Table 2). The finding indicates that the PoTup1–Cyc8 complex is the main interactor for PoCreA. On the basis of previous silver staining results and TAP-MS experiment for PoCreA, PoTup1 and PoCyc8 are considered as the putative interacting proteins of PoCreA under glucose condition.

**Table 2:** The top 10 proteins interacting with PoCreA identified through TAP-MS

Rank	Gene locus	emPAI^a^	*P. oxalicum* 114-2	*S. cerevisiae* S288C				Predicted function
			Protein	Homologue	Identity %	*E* value	Location^b^	
1st	PDE_03168	2.01 × 10^2^	PoCreA	Mig1	68	2e−29	Nucleus and cytoplasm	Sequence-specific DNA binding transcription factor involved in the regulation of transcription by RNA polymerase II in response to glucose and starvation
2nd	PDE_01024	11.18	PoTup1	Tup1	48	2e−128	Nucleus	General repressor of transcription, forms complex with Cyc8p
3rd	PDE_03177	2.16	PoCyc8	Cyc8	58	7e−149	Nucleus	General repressor of transcription, forms complex with Tup1p
4th	PDE_04157	1.22	–	–	–	–	–	Initiation-specific alpha-1,6-mannosyltransferase
5th	PDE_09900	0.74	–	Thi13	66	3e−175	Unknown	Protein involved in synthesis of the thiamine precursor HMP
6th	PDE_02746	0.50	–	–	–	–	–	Putative protein
7th	PDE_09681	0.49	–	Sps19	49	2e−87	Peroxisome	Peroxisomal 2,4-dienoyl-CoA reductase involved in fatty acid catabolism and sporulation
8th	PDE_07279	0.47	–	Atp2	79	0.0	Mitochondrion	Subunit of the catalytic core of the F1 sector of mitochondrial F1F0 ATP synthase
9th	PDE_04469	0.33	–	Cct2	74	0.0	Cytoplasm	Subunit of the chaperonin-containing T-complex (TriC) that mediates protein folding
10th	PDE_03408	0.32	–	Cdc19	66	0.0	Cytoplasm	Pyruvate kinase that catalyzes the final step in glycolysis, the conversion of phosphoenolpyruvate to pyruvate, which is then utilized in anaerobic or aerobic respiration

–: No significant similarity found

^a^emPAI is the Exponentially Modified Protein Abundance Index of three samples. Every Peptide count of each sample is listed in Additional file 3: Spreadsheet S1

^b^Data from Saccharomyces Genome Database (www.yeastgenome.org)

### PoCreA was observed in the protein–protein interaction of *P. oxalicum* PoCyc8

PoCyc8-TAP strain was constructed using the same method for *T. reesei*. The strains are listed in Additional file 1: Table S1. No significant difference in mycelia growth and conidia production was observed between the PoCyc8-TAP and the parent strain. In particular, their cellulolytic gene had a similar expression pattern (Additional file 2: Fig. S1C, D). Western blot analysis indicated the existence of PoCyc8 bait (Fig. 1E). Several specific bands were found between the PoCyc8-TAP and its parent strain 114-2 from the gel of SDS-PAGE with subsequent silver staining (Fig. 1F). The bands were cut from the gel and identified by LC–MS/MS as PoCyc8 (Fig. 1F, orange arrow, approximately 115 kDa, theoretical MW: 95.27 kDa) and PoTup1 (Fig. 1F, gray arrow, approximately 70 kDa; theoretical MW: 63.81 kDa). The proteins in the final eluent were identified by LC–MS/MS, and the TAP eluents from the parent strain *P. oxalicum* 114-2 were used as the control. The proteins in all three PoCyc8-TAP samples but not in any of the controls were considered putative interacting proteins.

In addition to PoCyc8 itself, 56 protein targets of putative interactions with PoCyc8 were captured (Additional file 3: Spreadsheet S1). The top 10 putative interacting protein targets with the highest emPAI are listed in Table 3. The top two proteins with the highest emPAI are PoCyc8 and PoTup1, thus verifying the stable interaction between PoTup1 and PoCyc8 and the consequent formation of the PoTup1–Cyc8 complex. Moreover, the 8th; 37th; 41st, and 45th positions are DNA-directed RNA Pol II subunit Rpb11; Rpb2 (the second largest subunit of Pol II); Rpb3 (the third largest subunit of Pol II), and Rpb1 (the largest subunit of Pol II), respectively (Additional file 3: Spreadsheet S1). PoCreA was also observed in the 50th position (Table 3, Additional file 3: Spreadsheet S1). This finding verified that PoCreA interacts with PoCyc8 in a direct or indirect way.

**Table 3:** The top 10 proteins interacting with PoCyc8 identified through TAP-MS

Rank	Gene locus	emPAI^a^	*P.oxalicum* 114-2	*S. cerevisiae* S288C				Predicted function
			Protein	Homologue	Identity %	*E* value	Location^b^	
1st	PDE_03177	2.63 × 10^11^	PoCyc8	Cyc8	58	7e−149	Nucleus	General repressor of transcription, forms complex with Tup1p
2nd	PDE_01024	1.39 × 10^8^	PoTup1	Tup1	48	2e−128	Nucleus	General repressor of transcription, forms complex with Cyc8p
3rd	PDE_02075	3.16 × 10^3^	–	Nfs1	74	0.0	Nucleus and mitochondrion	Mitochondrial cysteine desulfurase involved in iron-sulfur cluster assembly, tRNA thio-modification and tRNA wobble uridine modification; subunit of l-cysteine desulfurase complex
4th	PDE_05635	1.92 × 10^2^	–	Rpl38	49	2e−18	Cytoplasm and ribosome	Subunit of the cytosolic large ribosomal subunit; involved in translation
5th	PDE_08686	18.14	–	Vma2	85	0.0	Cytoplasm and vacuole membrane	Hydrogen ion transporting ATPase involved in vacuolar acidification, calcium homeostasis, and the assembly of proteasome storage granules
6th	PDE_05790	7.80	–	Rpt6	77	0.0	Nucleus and cytoplasm	Putative ATPase involved in proteasome regulatory particle assembly
7th	PDE_01647	6.80	–	Rpt1	77	0.0	Nucleus and cytoplasm	Putative ATPase involved in proteasome regulatory particle assembly
8th	PDE_00902	6.02	–	Rpb11	50	4e−38	Nucleus	DNA-directed RNA polymerase II subunit RPB11
9th	PDE_08458	5.45	–	Psa1	73	0.0	Nucleus and cytoplasm	Mannose-1-phosphate guanyltransferase; synthesizes GDP-mannose from GTP and mannose-1-phosphate in cell wall biosynthesis
10th	PDE_07279	5.39	–	Atp2	79	0.0	Mitochondrion	Subunit of the catalytic core of the F1 sector of mitochondrial F1F0 ATP synthase
……	……	……	……	……	……	……	……	……
50th	PDE_03168	0.35	PoCreA	Mig1	68	2e−29	Nucleus and cytoplasm	Sequence-specific DNA binding transcription factor involved in the regulation of transcription by RNA polymerase II in response to glucose and starvation

^a^emPAI is the Exponentially Modified Protein Abundance Index of three samples. Every Peptide count of each sample is listed in the Additional file 3: Spreadsheet S1

^b^Data from Saccharomyces Genome Database (www.yeastgenome.org)

–: No significant similarity found

### PoCreA physically interacts with the PoCyc8-Tup1 complex in the nucleus

TAP-MS results for *T. reesei* TrCRE1 and *P. oxalicum* PoCreA suggested that CRE1/CreA recruits the Tup1–Cyc8 complex. However, the putative interacting proteins identified by TAP-MS might include those that indirectly interact with CRE1/CreA as mediated by other proteins. In addition, the specific subunit of the complex that directly interacts with CRE1/CreA remains unknown. Whether Tup1 or Cyc8 mediates the interaction between CRE1/CreA and the complex must be investigated.

BiFC analysis [34] was used to determine (1) the real physical interaction between PoCreA and PoTup1–Cyc8 complex, and (2) the subunit of the complex that directly interacts with PoCreA. This method directly visualizes protein interactions in living cells. When two proteins gather together due to interaction, they carry two non-fluorescent fragments of yellow fluorescent protein (YFP) to complement each other, thus resulting in yellow fluorescence [35]. Several BiFC strains were constructed for the following analyses: PoCyc8-YFP-PoCreA strain to investigate the physical interaction between PoCreA and PoCyc8; PoTup1-YFP-PoCreA strain to investigate the physical interaction between PoCreA and PoTup1; and PoCyc8-YFP-empty, PoTup1-YFP-empty, and empty-YFP-empty strains as a negative control. No significant difference in mycelia growth and conidia production was observed between the BiFC strains and the parent strain. In particular, their cellulolytic genes had a similar expression pattern (Additional file 2: Fig. S1C, D). The construction strategies and strain verification are shown in Additional file 4: Fig. S2.

TAP-MS results for PoCyc8 suggested that TF PoCreA recruits the complex by interacting with the subunit PoCyc8, however, its interaction with PoTup1 is unverified. Yellow fluorescence was observed in the nucleus of PoCyc8-YFP-PoCreA and PoTup1-YFP-PoCreA BiFC strains (Fig. 1G, H) but not in any of the negative control BiFC strains (Additional file 2: Fig. S1E–G). These results suggest that PoCreA interacts with both PoTup1 and PoCyc8. The SWISS-MODEL SERVER [36] was then used to model PoTup1, PoCyc8, and PoCreA, respectively. The protein–protein docking between PoCreA and PoTup1–Cyc8 complex was predicted by the HDOCK SERVER [37]. The model with the highest score is shown in Additional file 5: Fig. S3. The putative model also supports the interaction of PoCreA with PoTup1 and PoCyc8.

### PoCreA affected the histone methylation patterns of H3K4 and H3K36

TAP-MS and BiFC results revealed that CRE1/CreA physically interacts with Tup1–Cyc8 in the nucleus. Therefore, the mechanism on how CRE1/CreA-Tup1–Cyc8 represses transcription must be determined. The initial hypothesis is that histone modification and chromatin structure change are the main mechanisms of the gene expression-inhibiting function of the complex [38]. Histone methylation, specifically on histone H3, regulates cellulolytic gene expression [39–42]. Even the expression of CRE1 itself is related to H3K4 methylation [43]. Whether CRE1/CreA-Tup1–Cyc8 is related to histone methylation and thus affects the transcription must be investigated. Two Po*creA* mutant strains including Po*creA* mutant ΔPo*creA* (ΔPo*creA*::*hph*) [8] and Po*creA* mutant OEPo*creA* (*ptrA*::*PgpdA*::Po*creA*) [16] were chosen for the investigation of the effect of CRE1/CreA-Tup1–Cyc8 interference on histone methylation.

**Fig. 2:**
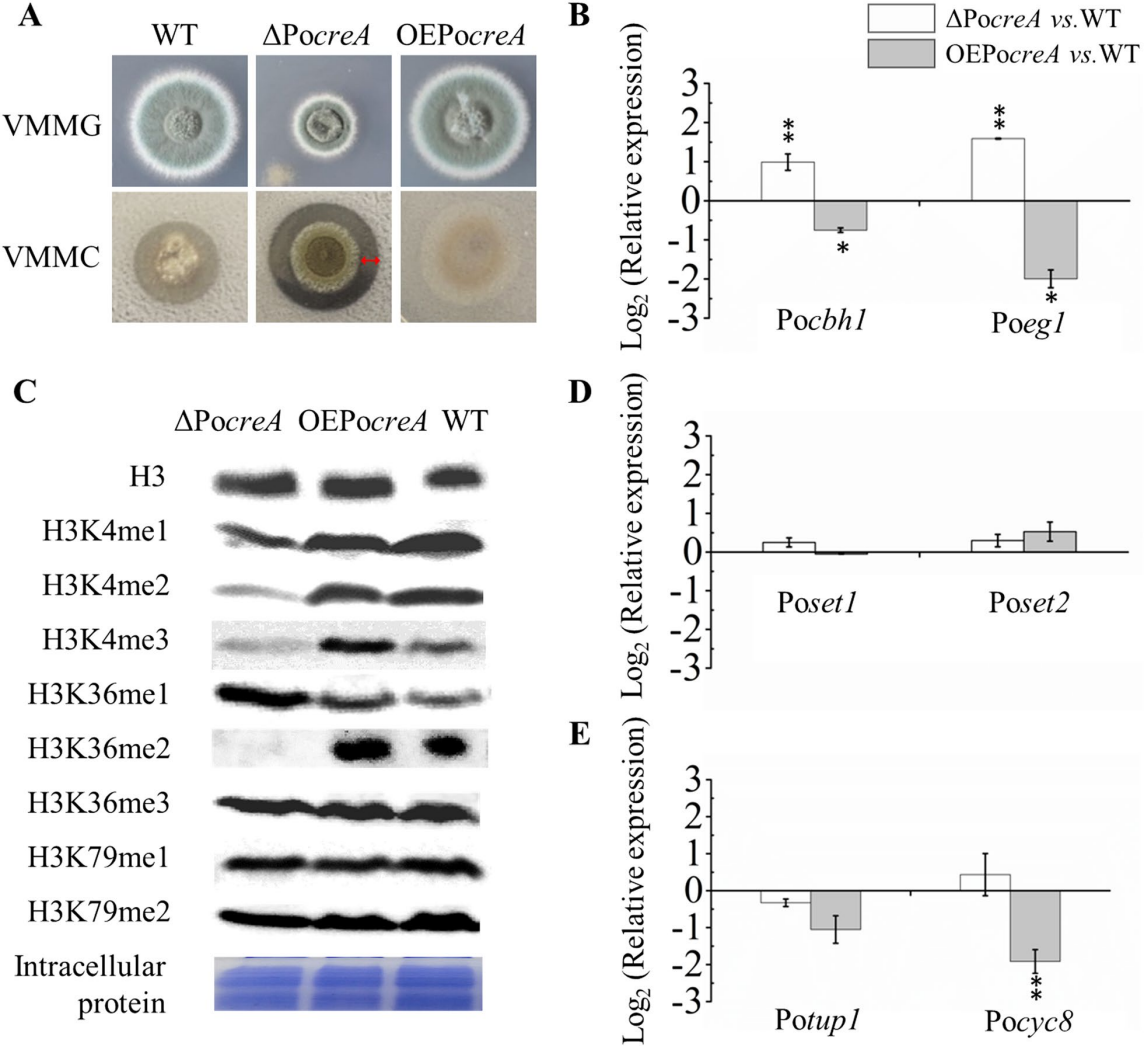
Analysis of histone methylation patterns and transcription levels of genes in *P. oxalicum* WT and mutants. **A** Observation of cellulolytic halo around the colonies, red arrow represents cellulolytic halo. **B** The transcript abundance of two prominent cellulase encoding genes, Po*cbh1* and Po*egl1*. **C** Assays of histone methylation patterns using Western blot. Histone H3 was used as the loading control. **D** The transcript abundance of two histone methyltransferases encoding genes, Po*set1* and Po*set2.* **E** The transcript abundance of genes Po*tup1* and Po*cyc8.* Statistical significance tests were performed by one tailed, unequal variance *t*-test. *P < 0.05, **P < 0.01, ***P < 0.001

First, the two mutants and the WT strain were cultivated on two different culture media: one is Vogel’s minimal medium (VMM) plus glucose (VMMG), a medium that represses the expression of cellulase and hemicellulase encoding genes during catabolite repression initiated by glucose [44], and the other is VMM plus cellulose (VMMC), a medium that activates the expression of cellulase and hemicellulase encoding genes, as the degradation products of cellulose, such as small amounts of cello-oligosaccharides, act as inducers [45]. The same amount (10^6^) fresh spores of WT, ΔPo*creA*, and OEPo*creA* were grown on VMMG or VMMC agar for 5 days. On VMMG agar, the ΔPo*creA* displayed diminished colony diameter. On VMMC agar, only the ΔPo*creA* mutant showed cellulolytic halo compared with the WT and OEPo*creA* mutant (Fig. 2A). This finding suggested that the ΔPo*creA* mutant secretes cellulolytic enzymes into the agar around the colony. The expression of two prominent cellulolytic genes, cellobiohydrolase-encoding gene Po*cbh1* (*cel7A*, PDE_07945) and endoglucanase-encoding gene Po*egl1* (*cel7B*, PDE_07929) was assayed after the strains were cultivated in VMMG liquid. The expression of gene Po*cbh1* and gene Po*egl1* was significantly upregulated in ΔPo*creA* but significantly downregulated in OEPo*creA*, in comparison to the WT (Fig. 2B). These results are consistent with previous reports and support the roles of PoCreA in CCR.

The mono-methylation (me1); di-methylation (me2), and tri-methylation (me3) of H3K4 (H3K4me1, H3K4me2, and H3K4me3) and H3K36 (H3K36me1, H3K36me2, and H3K36me3) and the mono-/di-methylation levels of H3K79 (H3K79me1 and H3K79me2) were also assayed when the strains were cultivated on in VMMG (Fig. 2C). ΔPo*creA* and OEPo*creA* mutants showed similar methylation patterns for H3K4me1, H3K36me1, H3K36me3, H3K79me1, and H3K79me2 compared with the WT. For the difference in the patterns of H3K4me3 and H3K36me2, the ΔPo*creA* mutant had a low level of H3K4me3 and no H3K36me2. Original Western blot images are shown in Additional file 6: Fig. S4.

Whether the dysregulation of PoCreA affects the expression of genes crucial for H3K4 and H3K36 methylation must be explored. Similar to *S. cerevisiae* having two methyltransferases Set1p and Set2p containing the evolutionarily conserved Su (var) 3–9, Enhancer-of-zeste, and Trithorax (SET) domain [46, 47], *P. oxalicum* also possesses two histone methyltransferase PoSet1 and PoSet2, which perform H3K4 and H3K36 methylation, respectively [40]. Therefore, the expression levels of Po*set1* and Po*set2* were investigated (Fig. 2D). The transcription of Po*set1* or Po*set2* did not show a difference (fold change < 2, *P* value > 0.05) in either ΔPo*creA* mutant or OEPo*creA* mutant compared with that in the WT (Fig. 2D). This finding suggested that the effect of PoCreA dysregulation on the methylation patterns of H3K4 and H3K36 is not due to its influence on the transcription of Po*set1* or Po*set2*. The expression of gene Po*tup1* and Po*cyc8* was also assayed and did not show a significant difference (fold change < 2, *P* value > 0.05) in the ΔPo*creA* mutant compared with that in the WT (Fig. 2E). This result suggested that the deletion of Po*creA* has no serious effect on the expression of these two genes. Po*cyc8* gene was downregulated in the OEPo*creA* strain; however, the exact reason is unknown.

### PoCyc8 directly interacts with PoSet2, and the level of histone H3K36me2 in the promoter of cellulolytic gene is positively correlated with PoCreA

**Fig. 3:**
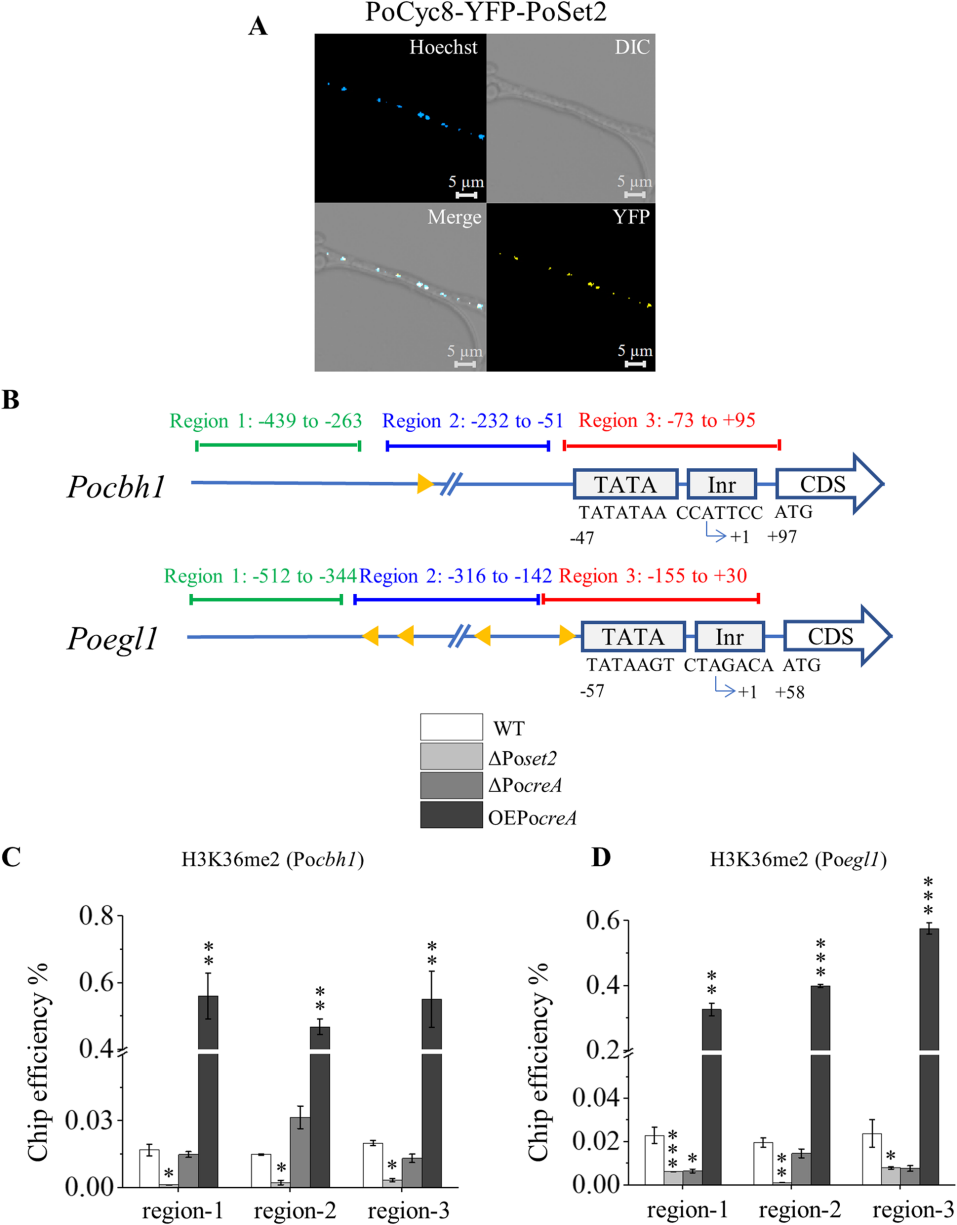
Strategy and results of ChIP-qPCR. **A** Microscopy of PoCyc8-YFP-PoSet2 BiFC strain. The image includes four parts. The upper left, blue particles indicate nucleus stained with Hoechst 33342. The upper right, normal white light. The bottom right, yellow fluorescent particles indicate interactions between two PoCyc8 and PoSet2. The bottom left, indicating the merge of yellow fluorescence and blue nucleus. **B** Overview on the upstream sequence and core promoters of Po*chb1* and Po*egl1*. The transcription start site (TSS) is designated as + 1. The initiator (Inr) and TATA box were illustrated. The three chromatin regions investigated by ChIP-qPCR are indicated by green, red, and blue bars, respectively. For Po*chb1*, region 1 covers from − 439 to − 263; region 2 covers from − 232 to − 51; region 3 covers from − 73 to + 95. For Po*egl1*, region 1 covers from − 512 to − 344; region 2 covers from − 316 to − 142; region 3 covers from − 155 to + 30. The putative DNA-binding sites of PoCreA (5′-GCGGAG-3′; 5′-CCGGGG-3′; 5′-CCCCGC-3′; 5′-CCCCGG-3′; 5′-CTCCGG-3′) are indicated by orange triangles. The orientation of the triangle represents the orientation of the binding motif. Inr, initiator element; TATA, TATA box. **C** ChIP-qPCR for H3K36me2 of Po*cbh1*. **D** ChIP-qPCR for H3K36me2 of Po*egl1*. All values are means from measurements in triplicates and three biological experiments. The error bars indicate standard deviations. *P < 0.05, **P < 0.01, ***P < 0.001

*Saccharomyces* Genome Database (SGD) verified that 51, 92, and 77 proteins physically interact with Mig1p, Tup1p, and Cyc8p, respectively. Among these proteins, histone methyltransferase Set2p physically interacts with Cyc8p [48]. Combined with the results of histone methylation patterns in Fig. 2C, this finding gave a hint that the complex Tup1–Cyc8 is a bridge between the TF and the histone methyltransferase Set2. Therefore, the BiFC strain PoCyc8-YFP-PoSet2 was constructed to investigate the physical interaction between PoCyc8 and PoSet2. Analysis of the direct protein–protein interaction between PoCyc8 and PoSet2 revealed yellow fluorescence localized in the nucleus (Fig. 3A) but not in any of the negative control BiFC strains (Additional file 2: Fig. S1E–G). This result indicated that the PoTup1–Cyc8 complex serves as a bridge for TF PoCreA and methyltransferase PoSet2.

Further analysis was conducted on the level of H3K36me2 in different regions upstream of the promoter of cellulase gene Po*cbh1* and Po*egl1* in mutants ΔPo*creA* and OEPo*creA*. The 5ʹ sequence of the promoters of Po*cbh1* and Po*egl1* was divided into three regions (Fig. 3B). The upstream of Po*cbh1* promoter included region 1 (− 439 to − 263), region 2 (− 232 to − 51), and region 3 (− 73 to + 95). The upstream of Po*egl1* promoter included region 1 (− 512 to − 344), region 2 (− 316 to − 142), and region 3 (− 155 to + 30). Region 3 covers the eukaryotic core promoter, the minimal set of sequence elements required for accurate transcription initiation by the Pol II machinery [49]. Initiator (Inr) and the TATA boxes were found in the core promoter region for Po*cbh1* and Po*egl1* (Fig. 3B)*.* Regions 1 and 2 were upstream of the core promoters and contain the binding sequences for gene-specific IFs. The consensus sequence for PoCreA binding is 5ʹ-SYGGRG-3ʹ [11, 50]. The Po*cbh1* bores the putative PoCreA-binding sites 5ʹ-GCGGAG-3ʹ distributed in region − 210 to − 205. The Po*egl1* bores the putative PoCreA-binding sites 5ʹ-CCGGGG-3ʹ, 5ʹ-CCCCGC-3ʹ, 5ʹ-CCCCGG-3ʹ, and 5ʹ-CTCCGG-3ʹ distributed in regions − 77 to − 72, − 237 to − 232, − 259 to − 254, and − 312 to − 307, respectively (Fig. 3B)*.*

ChIP-qPCR is performed to analyze histone modifications of target loci in the genome. In ChIP-qPCR, immune-enriched DNA fragments are identified and quantified. ChIP was performed using anti-H3K36me2 antibody, combined with qPCR to detect the levels of histone H3K36me2 in the promoter of cellulolytic genes Po*cbh1* and Po*egl1*. A remarkably increased level of H3K36me2 was observed in all the detected regions of Po*cbh1* and Po*egl1* for OEPo*creA.* In the ΔPo*creA*, a decreased level of H3K36me2 was observed in two (region 1 and region 3) of the three detected regions of Po*egl1* but not in any region of Po*cbh1* (Fig. 3C, D). The absence of PoCreA generally affected the low levels H3k36me2, but this influence was not significant. However, the overexpression of Po*creA* significantly increased the H3K36me2 level at specific cellulolytic gene loci. The reduced level of H3K36me2 in ΔPo*creA* and the increased level of H3K36me2 in OEPo*creA* implied that PoCreA is positively correlated with H3K36me2 level. In addition, the deletion of Po*set2* (ΔPo*set2*) [40], showed significantly decreased H3K36me2 levels in the three regions of Po*cbh1* and Po*egl1*, indicating that H3K36me2 is mainly mediated by PoSet2 (Fig. 3C, D). Therefore, PoCreA recruits PoTup1–Cyc8. Histone methyltransferase PoSet2 is also involved in the regulatory network via its interaction with PoCyc8. Given that PoSet2 and H3K36me2 on the promoter of cellulolytic genes are the repression marker of the target cellulolytic genes [40], the gene is inactivated by PoCreA-Tup1-Cyc8-Set2-mediated repression.

## Discussion

CCR is a general phenomenon in various bacteria, yeast, filamentous fungi, and other microorganisms. The presence of carbon sources e.g., glucose and related sugars represses the transcription of certain genes. As a sequence-specific TF, CRE1/CreA plays a central role in CCR and is essential for the adaptation and survival of several species, such as *Aspergillus*, *Penicillium*, and *Trichoderma* [8, 13, 15]. In *T. reesei*, TrCRE1 rapidly shifts from cytoplasmic to nuclear with glucose addition [14] and represses the expression of glucose-repressible cellulolytic genes (such as *cbh2* or *egl1*) [51]. Whether TrCRE1 binding ultimately regulates transcription upon DNA binding remains unclear.

In general, eukaryotic TFs regulate transcription without directly interacting with RNA Pol II but through recruiting cofactors that promote (or hinder) specific phases of transcription [52, 53]. The cofactors might be “coactivators” or “corepressors”—usually large multi-subunit protein complexes that regulate transcription via several different mechanisms. TAP-MS results for TrCRE1 and PoCreA, showed that as a homologue of yeast Mig1p, TrCRE1 and PoCreA might recruit the Tup1–Cyc8 complex involved in gene repression. These results verify the long-standing conjecture in the research field of filamentous fungi that CRE1/CreA recruits the corepressor complex Tup1–Cyc8 to participate in gene expression and CCR. In addition to CRE1/CreA and Tup1–Cyc8, other regulators such as CreB, CreC, and CreD also participate in CCR in *A. nidulans* [54]. However, the homologues of these proteins have not been identified from the putative interaction proteins of TrCRE1 or PoCreA. Alam et al. also reported the lack of direct physical interaction between CreA and CreB [54], which can be explained by two reasons. First, CreA does not directly interact with CreB. Second, the affinity of their direct interaction is low and was not detected due to the limitation of experimental technology.

More putative protein targets exhibit putative interactions with PoCyc8 than with PoCreA (Additional file 3: Spreadsheet S1). PoCyc8 and TrCYC8 are orthologs of *S. cerevisiae* Cyc8 and share 55% and 58% identity with the sequence of *S. cerevisiae* Cyc8, respectively. *S. cerevisiae* Cyc8p and PoCyc8 possess 10 copies of the 34-amino-acid tetratricopeptide repeat (TPR) motifs, and TrCYC8 possesses 9 copies of TPR (Additional file 7: Fig. S5A). TRP motifs form a helix-turn-helix arrangement and provide a structural scaffold for the mediation of multiple protein–protein interactions [55]. This finding explains the higher number of proteins interacting with the PoCyc8 than with PoCreA. In addition, the deletion of PoCyc8 is lethal in *P. oxalicum*, implying that the complex has more extensive regulatory roles than TF PoCreA.

The Tup1–Cyc8 complex is a conserved corepressor of transcriptional expression in eukaryotes. TrTUP1 and PoTup1 are orthologs of *S. cerevisiae* Tup1p, and share 49% and 48% identity with the sequence of *S. cerevisiae* Tup1, respectively. All of them possess seven highly conserved repeat WD40 domains (Additional file 7: Fig. S5B). These genes are single copy in *T. reesei* and *P. oxalicum*. In *S. cerevisiae*, the Tup1p-Cyc8 complex is composed of four Tup1 and one Cyc8p subunit [56]. However, TAP-MS results for PoCyc8-TAP revealed that the emPAI of PoTup1 was lower than that of PoCyc8 (Table 3). Additional experimental evidence is needed to define the proportion of Tup1 and Cyc8 in the complex.

Although the mechanism for gene repression by the Tup1–Cyc8 complex in filamentous fungi remains poorly understood, several working models of Tup1–Cyc8 regulation in yeast have been proposed, including the interaction with histone deacetylases and modification of chromatin structures, interaction with the general transcription machinery, and blocking the activation domains of transcriptional activators [57]. This work showed that the mechanisms of turning genes off by PoCreA-Tup1–Cyc8 in cellulolytic filamentous fungi exhibited similarities and differences with those in yeast.

*Trichoderma reesei* TrCRE1 was confirmed to be indirectly related to the change of chromatin structure, specifically on the promoter region of the cellulolytic genes; however, the exact reason is not known [10, 58]. In *A. nidulans*, the deletion of the RcoA (the homologue of Tup1p) alters the chromatin structure of promoters for carbon catabolite repressible genes *alcA*, *alcR*, and *prnD–prnB* [27]. Therefore, CRE1/CreA-Tup1–Cyc8 might interact with some proteins related to chromatin modification, such as histone-modifying enzymes or chromatin-remodeling complexes. Direct interaction was observed between PoCyc8 and histone methyltransferase PoSet2, suggesting that the PoTup1–Cyc8 complex bridges the TF PoCreA and histone methyltransferase PoSet2. The transcription of Po*set1* or Po*set2* did not differ in either ΔPo*creA* mutant or OEPo*creA* mutant compared with that in the WT (Fig. 2D). Similar results were obtained from the analysis on the effects of CRE1/CreA on the transcription of *set1*, *set2*, *tup1*, and *cyc8* in other filamentous fungi according to their transcriptome data (Additional file 8: Fig. S6). Analysis was conducted on the data obtained for *T. reesei* Tr*cre1* deletion strain (GEO accession: GSE57374) [5] and *Magnaporthe grisea* Mg*cre1* deletion strain (GEO accession: GSE153084) [59] cultivated under glucose condition. In the *cre1* deletion strains, the expression levels of *set1* gene (homologue ID 81925 in *T. reesei* and MGG_15053 in *M. grisea*), *set2* gene (homologue ID 80732 in *T. reesei* and MGG_01661 in *M. grisea*), *tup1* gene (homologue ID 121940 in *T. reesei* and MGG_08829 in *M. grisea*), and *cyc8* gene (homologue ID 102616 in *T. reesei* and MGG_03196 in *M. grisea*) in the mutants were not different from those of their corresponding parent strains, with the fold changes for transcripts < 2 (Additional file 8: Fig. S6). These results suggested that CRE1/CreA does not directly affect the expression of the above genes. *P. oxalicum* PoCreA possibly affects histone methylation through other mechanisms.

In *S. cerevisiae*, Set2p physically interacts with Cyc8p [48]. Although the deletion of Set2p in yeast does not affect the Tup1-Cyc8-mediated repression of well-defined targets [60], our previous study showed that the deletion of *P. oxalicum* Po*set2* upregulated the transcription of cellulolytic genes accompanied by a decrease in H3K36 methylation on specific cellulolytic gene loci [40]. Meanwhile, Po*set2* overexpression downregulated the transcription of cellulolytic genes accompanied by changes in the chromatin structure around the promoter and transcription start site (TSS) [40]. In the present study, the level of histone H3K36me2 in the promoter of cellulolytic genes was found to be positively correlated with PoCreA protein levels. Therefore, a high amount of PoCreA protein presumably recruits a high amount of PoTup1–Cyc8 complex and PoSet2, followed by a high level of methylation of H3K36, a change in the local chromatin environment, and repressed cellulolytic genes.

In the regulation of PoCreA-Tup1–Cyc8-Set2, H3K36 methylation was discovered as a repression marker for cellulolytic gene transcription. This result is unexpected because Set2p is commonly associated with transcriptional activation [46, 61]. However, many reports supported the important role of Set2p in gene repression. For example, approximately 80 mRNA genes in yeast were activated upon Set2p absence [62]. Set2p also prevents transcription initiation by recruiting a repressive histone deacetylase (HDAC) Rpd3S complex to change the chromatin structures after Pol II passage, thereby suppressing transcription initiation and slowing down elongation [38, 63]. Meanwhile, yeast Tup1p–Cyc8p repression functions are always linked to the changes in chromatin structure mediated by recruiting Rpd3S complex [64], which supports the relation between the complex Tup1p–Cyc8p and Set2p.

In yeast, the gene repression effect of corepressor complex Tup1–Cyc8 is also related to the general transcription machinery. Once at the promoter, the complex Tup1–Cyc8(Ssn6) interacts with mediator subunits, such as SIN4/MED16, Hrs1/MED3, and SRB7/MED21, thus preventing DNA-directed Pol II holoenzyme to be recruited to the core promoter or halt transcription initiation [65, 66]. However, no mediator subunit was found in the results of PoCyc8-TAP, although the PoTup1–Cyc8 complex was evident. Four Pol II subunits, namely, Rpb1, Rpb2, Rpb3, and Rpb11 were observed. Rpb1 and Rpb2, as the largest and second-largest catalytic subunits of RNA Pol II, together with third-largest subunit Rpb3, and Rpb10, Rpb11, Rpb12 subunits, form the central large cleft, which is the polymerase active center [67]. Yeast Cyc8p also directly interacts with Rpb3p as revealed by Affinity Capture-Western assay [68]. Therefore, another hypothesis for these results is that PoCreA-Cyc8–Tup1-mediated repression occurs via direct interaction with some components of the Pol II (possibly subunit Rpb3) and hinders the Pol II from progressing downstream of the promoter. However, it would necessitate further research to confirm this hypothesis.

**Fig. 4:**
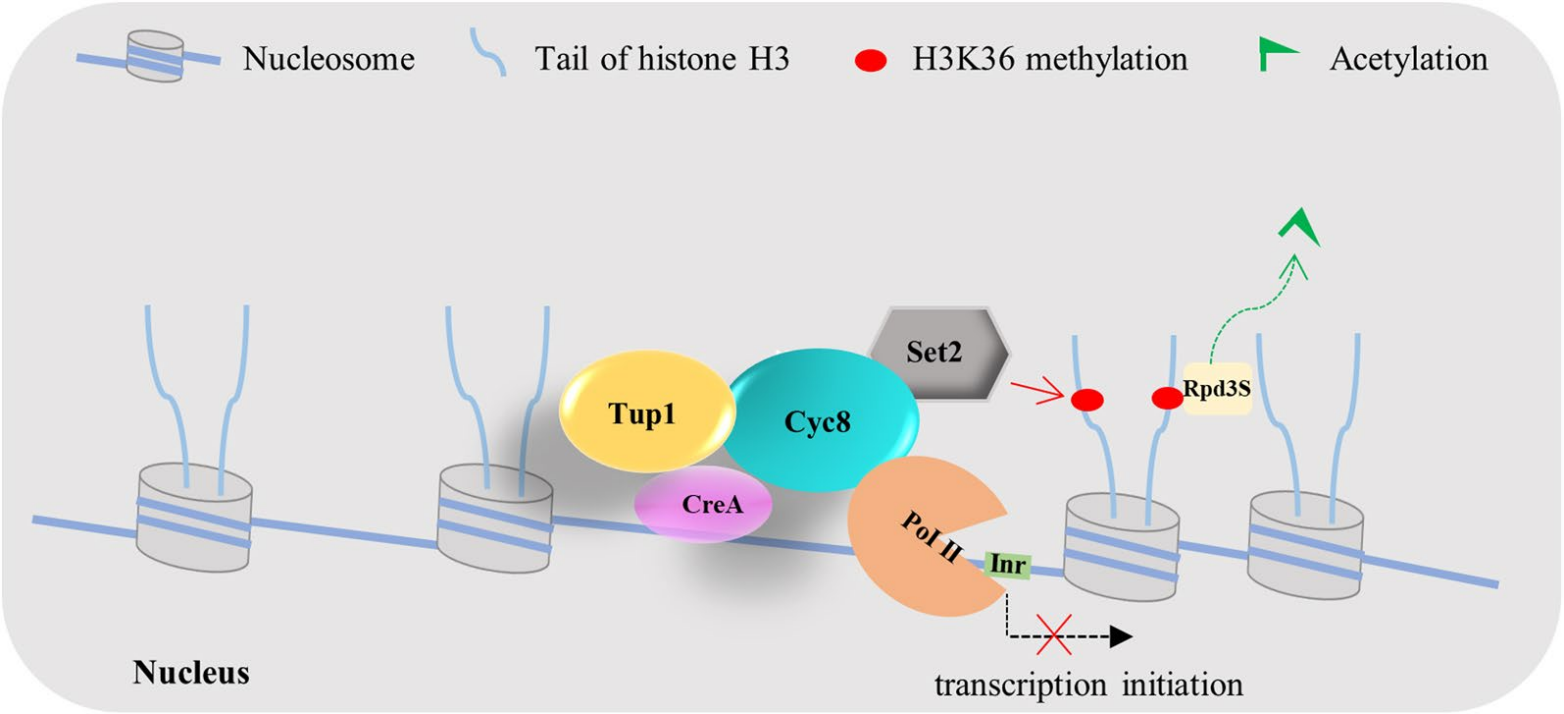
The model for PoCreA-CYC8–TUP1 during repression of cellulolytic gene. In glucose condition, PoCreA mainly localizes in the nucleus, binds to the promoter of the target genes, and recruits the corepressor complex PoTup1–Cyc8. Histone methyltransferase Set2 which methylates H3K36, is also involved in the regulatory network via its interaction with Cyc8. As the repression marker of cellulolytic gene expression, H3K36 methylation together with histone deacetylase Rpd3 cooperate to reestablish chromatin, thereby suppresses inappropriate transcription initiation. In addition, the corepressor PoTup1–Cyc8 also interacts with the main subunit of the RNA Pol II and thus prevents Pol II from initiating transcription

Although no direct evidence confirms that TF CRE1/CreA, complex Tup1-Cyc8, RNA Pol II and histone methyltransferase Set2 co-occupy on the promoter, a new research in yeast supported that Tup1p, RNA Pol II (Rpb3p), and Set2p occupy near the TSS [69]. On the basis of previous reports and present data, a model for PoCreA-Tup1–Cyc8 during the repression of the cellulolytic gene was proposed (Fig. 4). In the presence of glucose, PoCreA mainly localizes in the nucleus, binds to the promoter of the target genes, and recruits the corepressor complex PoTup1–Cyc8. Histone methyltransferase Set2, which methylates H3K36, is also involved in the regulatory network by interacting with PoCyc8. As the repression marker of cellulolytic gene expression, H3K36 methylation and histone deacetylase Rpd3 cooperate to reestablish chromatin, thereby suppressing inappropriate transcription initiation. In addition, the corepressor PoTup1–Cyc8 also interacts with the main subunit of the RNA Pol II and thus prevents Pol II from initiating transcription (Fig. 4). It is noting the model is mainly applicable to *P. oxalicum*, although the interaction between CRE1/CreA and co-repressor complex Cyc8–Tup1 is conservative.

## Conclusions

This study verifies the long-standing conjecture that TF CRE1/CreA represses gene expression via interacting with the corepressor complex Tup1–Cyc8 in two cellulase-producers *T. reesei* and *P*. *oxalicum*. An explanation that the cellulolytic gene is repressed by PoCreA-Tup1-Cyc8-Set2-mediated transcriptional repression in *P*. *oxalicum*, was presented. The findings contribute to the understanding of CCR mechanism in filamentous fungi and serve as a guide for biotechnologically relevant enzyme production.

## Material and methods

### Strains and culture condition

The WT strain *P. oxalicum* 114-2 (CGMCC 5302) and the mutants ΔPo*creA* and OEPo*creA* [8, 16] were cultivated on 10% wheat bran extract agar slants at 30 °C for 5 days. *T. reesei* QP4 [31] was cultivated on potato dextrose agar (PDA) with Vogel’s minimal medium (VMM) [70] agar added with 2% glucose (VMMG) at 30 °C for 5 days. All strains used in this study are listed in Additional file 1: Table S1.

### Construction of strains for TAP and BiFC

For TAP strains, the homologous recombination was used to knock-in FLAG and HA tags before the C-terminal stop codon of the bait protein. The strategy of TAP strains construction is shown in Additional file 4: Fig. S2A. To construct the TrCRE1-TAP strain (TrCRE1-FLAG-HA) in *T. reesei* QP4: primers TrCRE1-F/TrCRE1-*tap*-R were used to amplify the upstream homologous arm (1585 bp) of the gene Tr*cre1*. Primers TrCRE1*-*DF/TrCRE1*-*DR were used to amplify the downstream homologous arm (1667 bp) of the gene Tr*cre1*. Primers *pyrG-*F/*pyrG-*R were used to amplify the marker gene *pyrG* (1434 bp) from the genome of *A. nidulans*. The upstream homologous arm, *pyrG* gene, and downstream homologous arm were fused by overlapping PCR and then amplified by nested primers TrCRE1-CSF/TrCRE1*-*CSR. The fused PCR product (4464 bp) was transformed into *T. reesei* QP4 through polyethylene glycol (PEG)-mediated protoplast transformation [71] to obtain the TAP strain TrCRE1-TAP. The same method was applied to construct *P. oxalicum* PoCreA-TAP strain (PoCreA-FLAG-HA), and PoCyc8-TAP strain (PoCyc8-FLAG-HA). Primers PoCreA-F/PoCreA-*tap*-R and PoCyc8-F/PoCyc8-*tap*-R were used to amplify the upstream homologous arms of the gene Po*creA* and Po*cyc8* (2109 and 3466 bp, respectively). Primers PoCreA*-*DF/PoCreA*-*DR and PoCyc8*-*DF/PoCyc8*-*DR were used to amplify the downstream homologous arms of gene Po*creA* and Po*cyc8* (1998 and 1521 bp, respectively). Primers *hygA-*F/*hygA-*R were used to amplify the marker hygromycin gene *hygA* (1954 bp) from the template of plasmid pSilent1 [72]. The upstream homologous sequence, *hygA* gene and downstream homologous sequence were fused by overlapping PCR and then amplified by nested primers PoCreA-CSF/PoCreA*-*CSR and PoCyc8-CSF/PoCyc8*-*CSR, respectively. The two fused PCR products (5275 and 6752 bp) were transformed into *P. oxalicum* 114-2 using PEG-mediated protoplast transformation [71] to obtain the TAP strains PoCreA-TAP, and PoCyc8-TAP, respectively. The primers used for PCR amplification are listed in Additional file 9: Table S2.

BiFC strains were constructed as previously described [34], and the strategy of construction is shown in Additional file 4: Fig. S2. The plasmid pMD18-T-NYFP carries the encoding sequence of N-terminal (1–155 aa) of the yellow fluorescent protein (YFP) (Additional file 4: Fig. S2C), and the plasmid pUC19-CYFP carries the encoding sequence of C-terminal (156–238 aa) of the YFP (Additional file 4: Fig. S2D). Primers PoTup1-NF/PoTup1-NR and PoCyc8-NF/PoCyc8-NR were used to amplify the Po*tup1* and Po*cyc8* genes, respectively, which were inserted into the multiple cloning site (MCS) of pMD18-T-NYFP to obtain the recombined pMD18-T-NYFP-PoTup1 and pMD18-T-NYFP-PoCyc8 vectors, respectively. Similarly, the primers PoCreA-CF/PoCreA-CR and PoSet2-CF/PoSet2-CR were used to amplify the genes Po*creA* and Po*set2*, which were then inserted into the MCS of pUC19-CYFP to obtain the recombined pUC19-CYFP-PoCreA and pUC19-CYFP-PoSet2 vectors, respectively. Vectors pMD18-T-NYFP-PoTup1 and pUC19-CYFP-PoCreA were simultaneously transformed into the parent strain 114-2 to study the interaction between PoTup1 and PoCreA. Vectors pMD18-T-NYFP-PoCyc8 and pUC19-CYFP-PoCreA were simultaneously transformed into the parent strain 114-2 to study the interaction between PoCyc8 and PoCreA. Vectors pMD18-T-NYFP-PoCyc8 and pUC19-CYFP-PoSet2 were simultaneously transformed into the parent strain 114-2 to study the interaction between PoCyc8 and PoSet2. Similar method was used to construct negative control strains, namely, PoTup1-YFP-empty, PoCyc8-YFP-empty, and empty-YFP-empty. Two pairs of primers NYZF/NYZR and CYCF/CYZR were used to verify the BiFC strains (Additional file 4: Fig. S2E). The primers used for PCR amplification are listed in Additional file 9: Table S2.

### Phenotypic analysis and enzyme activity determination

For phenotype analysis, the fresh spore suspension was diluted to the same concentration (10^6^ conidia/mL). 1 μL of spore suspension were spotted on VMMG agar at 30 °C for 5 days. For enzyme activity assay, fresh spore suspensions of the parent and mutant strains were cultivated in VMMG liquid for 24 h. Afterward, 0.3 g of filtered hyphae was transferred to 100 mL of VMM added with 1% bran juice and 1% cellulose (w/v) media and mixed at 180 rpm and 30 °C. The filter paper enzyme activities (FPA) of the culture supernatants were assayed using DNS reagent [73]. Whatman No. 1 filter paper (GE Healthcare companies, UK) was applied as the substrate. One enzyme activity unit is defined as the amount of enzyme that can convert 1 μmol of the substrate in 1 min under the assay conditions.

### Microscopy of BiFC strains

Fresh spore suspensions of BiFC strains PoTup1-YFP-PoCreA; PoCyc8-YFP-PoCreA; PoCyc8-YFP-PoSet2 and negative control strains PoTup1-YFP-empty; PoCyc8-YFP-empty; empty-YFP-empty were spread on VMMG agar. Then, 18 mm sterile coverslips were inserted into the agar at a 45° angle. The cultures were incubated at 30 °C for 24 h. Hoechst 33342 (Sigma-Aldrich, United States) was used for nucleus staining. The blue nucleus stained by Hoechst 33342 was observed under 405 nm excitation light. Yellow fluorescence was observed by excitation light at 488 nm using the laser scanning confocal microscope (ZEISS LSM900) (Carl Zeiss).

### Protein–protein docking and domain architecture analysis

The SWISS-MODEL SERVE [36] was used to model target proteins. The 3D protein model was automatically generated by inputting the amino acid sequence of the target protein. The highest-scoring protein models of PoCreA, PoCyc8, and PoTup1 were individually created. The HDOCK SERVER [37] was then used to predict the protein–protein docking model. First, the models of PoCyc8 and PoTup1 were inputted as the receptor and ligand, respectively, to obtain the highest-scoring PoTup1/Cyc8 docking model. The models of PoTup1/Cyc8 and PoCreA were then inputted as the receptor and ligand, respectively, to obtain the PoCreA-Tup1/Cyc8 docking model.

### Total RNA extraction and gene expression analysis by qRT-PCR

The fresh spore suspensions of parent strain 114–2 and mutants ΔPo*creA* and OEPo*creA* were cultivated in VMMG liquid for 24 h. The mycelia were collected and ground in liquid nitrogen, and 100 mg of ground powder was then transferred into 1 mL of TRIzol reagent (TaKaRa Biotechnology). Total RNA extraction was performed in accordance with the manufacturer’s instructions. cDNA was obtained by PrimeScript RT Reagent kit with gDNA Eraser (TaKaRa Biotechnology). Three biological triplicates of qPCR assay of each gene were performed. Light Cycler 480 system with software version 4.0 (Roche, Mannheim, Germany) was used to perform the reaction procedure. The primers of expression of the specific gene Po*cbh1*, Po*egl1*, Po*set1*, Po*set2*, Po*tup1*, Po*cyc8*, and Po*actin* assayed by qPCR are as follows: qPo*cbh1*F/qPo*cbh1*R; qPo*egl1*F/qPo*egl1*R; qPo*set1*F/qPo*set1*R; qPo*set2*F/qPo*set2*R; qPo*tup1*F/qPo*tup1*R; qPo*cyc8*F/qPo*cyc8*R; and qPo*actin*F/qPo*actin*R. The expression level of a specific gene is based on the control gene Po*actin* (PDE_01092). The outcome of relative expression of the examined gene was calculated as follows: copy number of target gene/actin gene. Statistical significance was considered at *P* ≤ 0.05. The primers used for qPCR are listed in Additional file 9: Table S2.

### Protein extraction and Western blot analysis

The fresh spore suspensions of parent strain 114-2 and mutants ΔPo*creA* and OEPo*creA* were cultivated in VMMG liquid for 24 h. The mycelia were collected and ground in liquid nitrogen, and 100 mg of ground powder was transferred into 200 μL of extraction buffer (per liter: 1 M pH7.5 Tris-HCl 50 mL, NaCl 8.76 g, NP-40 10 mL, 100 mM phenylmethanesulfonyl fluoride (PMSF) 10 mL). The samples were vigorously mixed by a vortex shaker, placed in an ice bath for 30 min, and centrifuged at 4 °C, 12,000 rpm for 10 min to obtain the supernatant. Protein concentrations in the supernatant were assayed using the Bradford method [74]. Equal amounts (200 ng) of total protein were separated by SDS polyacrylamide gel electrophoresis (SDS-PAGE) and then transferred to nitrocellulose membrane (Pall Corp., Ann Arbor, MI, United States) using a Bio-Rad electroblotting apparatus. The anti-H3K4me1 antibody (ab8895, Abcam, Cambridge, UK), anti-H3K4me2 antibody (A2356, ABclonal, Wuhan, China), and anti-H3K4me3 antibody (ab8580, Abcam, Cambridge, UK) were used to detect H3K4 methylation. The anti-H3K36me1 antibody (OM256826, OmnimAbs, California, USA), anti-H3K36me2 antibody (A2365, ABclonal, Wuhan, China), and anti-H3K36me3 antibody (ab9050, Abcam, Cambridge, UK) were used to detect H3K36 methylation. The anti-H3K79me1 antibody (OM256854, OmnimAbs, California, USA) and anti-H3K79me2 antibody (ab3594, Abcam, United Kingdom) were used to detect H3K79 methylation. Equal amounts of the total protein and the anti-histone H3 antibody (OM256785, OmnimAbs, California, USA) were set as the loading control. Western blot was performed following the detailed method described in [40].

### Chromatin immunoprecipitation and qRT-PCR (ChIP-qPCR) assay

Fresh spore suspensions of parent strain 114-2 and mutants ΔPo*creA* and OEPo*creA* were inoculated in VMMG liquid for 24 h and then added with 37% formaldehyde to crosslink the samples for 10 min and finally with 1.25M glycine to terminate the crosslink procedure. Pre-cooled TBS buffer was used to wash the mycelia, which were then drained and ground with liquid nitrogen. An appropriate amount of Chip-lysis buffer was added to lyse the ground mycelia to obtain the supernatant through centrifugation. The supernatant was separated through sonication with the condition of 10 s on and 10 s off for 72 cycles on ice to ensure that the chromatin was broken to 100–1000 bp. Afterward, 20 μL of blocked protein G/A beads (Thermo Fisher Scientific, MA, United States) were added in per 1.1 mL of the disrupted solution and stored in 4 °C for 4 h. In brief, 100 μL of the sample was obtained, labeled as input, and added with 1 μL of anti-H3K36me2 antibody to react overnight. Afterward, 50 μL of protein G/A beads were added to incubate for 4 h. Finally, the beads were eluted and de-crosslinked overnight with 20 μL of 5 M NaCl at 65 °C. DNA was extracted with phenol/chloroform/isoamyl alcohol. ChIP-enriched genomic DNA fragments were assayed by qPCR analysis using the following primers: Po*cbh1*-1F/Po*cbh1*-1R; Po*cbh1*-2F/Po*cbh1*-2R; Po*cbh1*-3F/Po*cbh1*-3R, and Po*egl1*-1F/Po*egl1*-1R; Po*egl1*-2F/Po*egl1*-2R; Po*egl1*-3F/Po*egl1*-3R. The relative enrichment of IP DNA was calculated by the input % method as follows (Ct = the number of cycles required to reach the threshold): ChIP efficiency = 2^−∆Ct^ × 100%, ∆Ct = Ct_IP_ − (Ct_Input_ − log_2_10) [40]. Three biological replicate experiments were performed for each strain. Statistical significance was considered at *P* ≤ 0.05. The primers used for ChIP-qPCR are listed in Additional file 9: Table S2.

### TAP and mass spectrometry

Fresh spore suspensions of the parent strain *P. oxalicum* 114-2, PoCreA-TAP, PoCyc8-TAP, *T. reesei* QP4 and TrCRE1-TAP strain were inoculated in 2 L of VMM liquid added with 2% glucose (VMMG) as a carbon source at 180 rpm for 24 h, at 30 °C in a shaker. The hyphae were filtered and washed by distilled water twice, ground with liquid nitrogen, transferred to a 100 mL centrifuge tube, and added with 15 mL of protein lysis buffer (NaCl 9 g, 1M Tris-HCl, pH 7.5, glycerin 100 mL, and NP40 1 mL, per 1 L) and 0.05% protease inhibitor cocktail. The samples were then centrifuged at 12,000 rpm and 4 °C for 30 min to obtain the suspension. For the first-step affinity purification, ANTI-FLAG M2 affinity resin (Sigma-Aldrich, United States) was added to the suspension and incubated overnight at 4 °C with rotation. The protein suspension was then centrifuged at 3000 rpm for 2 min at 4 °C to discard the supernatant. ANTI-FLAG M2 affinity resin was transferred to the spin columns and centrifuged at 3000 rpm for 30 s at 4 °C to discard the filtrate. Afterward, 500 μL of 3× FLAG peptide (final concentration 150 ng/μL) (Sigma-Aldrich, United States) was added to the spin columns and centrifuged at 3000 rpm for 1 min to obtain the first-step eluent. For the second-step affinity purification, the ANTI-HA resin (Thermo Fisher Scientific, MA, United States) was transferred to the first-step eluent, incubated at 4 °C for 2 h, transferred to the spin columns, and centrifuged at 3000 rpm for 30 s at 4 °C to discard the filtrate. Finally, 80 μL of 8 M urea was added and incubated with the ANTI-HA resin for 15 min. The spin columns were centrifuged at 3000 rpm for 1 min to obtain the final eluent, which was then divided into three parts: one for Western blot using the ANTI-HA antibody (ABclonal, China), one was separated by 12.5% SDS-PAGE and stained with silver reagent [75], and the last one was assayed through LC–MS/MS (APT, Shanghai, China) to determine the putative interacting proteins from the bait proteins. Exponentially modified protein abundance index (emPAI) was used for estimation of absolute protein amount using the following formula. Where *N*_observed_ is the number of experimentally observed peptides, and *N*_observable_ is the number of theoretically observable tryptic peptides for each protein. PAI = *N*_observed_/*N*_observable_, emPAI = 10^PAI^ – 1 [32].

## Supplementary Information

**Additional file 1: Table S1.** The strains used in this study.

**Additional file 2: Figure S1.** Phenotypic analysis and enzyme activity determination of TAP and BiFC strains. (A) Growth phenotype of TrCRE1-TAP strain and parent *T. reesei *QP4. (B) FPA activities assay of TrCRE1-TAP strain and parent* T. reesei *QP4. (C) Phenotypic analysis of TAP and BiFC strains in *P. oxalicum. *(D) FPA activities assay of TAP and BiFC strains in *P. oxalicum*. (E) Microscopy of PoTup1-YFP-empty BiFC strain. (F) Microscopy of PoCyc8-YFP-empty BiFC strain. (G) Microscopy of empty-YFP-empty BiFC strain.

**Additional file 3: Spreadsheet S1.** Proteins interacting with *T. reesei* TrCRE1, *P. oxalicum* PoCreA, and PoCyc8 identified through TAP-MS. The peptide counts (PepCount) of each biological replicate, the sum of PepCount of three biological replicates, the number of observable peptides, and Exponentially Modified Protein Abundance Index (emPAI) were listed. The proteins are arranged according to the value of emPAI.

**Additional file 4: Figure S2.** Construction strategy and verification of TAP and BiFC strains. (A) Construction strategy of TAP strains. (B) Results of diagnostic PCR of TAP strains. Lane 1 (1895 bp) and Lane 2 (1928 bp) represent TrCRE1-TAP (amplified using primers TrCRE1-F/pyrG-YZR and pyrG-YZF/TrCRE1-DR, respectively); lane 3 and Lane 4 represent negative control (*T. reesei* QP4); lane 5 (2289 bp) and Lane 6 (2234 bp) represent PoCreA-TAP (amplified using primers PoCreA-F/hygA-YZR and hygA-YZF/PoCreA-DR respectively); lane 7 and lane 8 represent negative control (*P. oxalicum* 114-2); lane 9 (3646 bp) and lane 10 represent PoCyc8-TAP (amplified using primers PoCyc8-F/hygA-YZR and hygA-YZF/PoCyc8-DR respectively); lane 11 and lane 12 represent negative control (*P. oxalicum* 114-2). The PCR products were sequenced to verify the proper insertion of FLAG-HA tags. (C) Map of pMD18-T-NYFP which carries the N-terminal (1–155 aa) of the YFP. (D) Map of pUC19-NYFP which carries C-terminal (156–238 aa) of the YFP. (E) Results of diagnostic PCR of BiFC strains using primers NYZF/NYZR (lane 1, 3, 5, 7, 9, 11, 13) and CYZF/CYZR (lane 2, 4, 6, 8, 10, 12, 14). Lane 1 (5200 bp) and lane 2 (2740 bp) represent PoCyc8-YFP-PoCreA; lane 3 (5200 bp) and lane 4 (4789 bp) represent PoCyc8-YFP-PoSet2; lane 5 (5217 bp) and lane 6 (2740 bp) represent PoTup1-YFP-PoCreA; lane 7 (5200 bp) and lane 8 (1515 bp) represent PoCyc8-YFP-empty; lane 9 (5217 bp) and lane 10 (1515 bp) represent PoTup1-YFP-empty; lane 11 (2271 bp) and lane 12 (1515 bp) represent empty-YFP-empty; lane 13 and 14 were negative control amplified by template of the parent strain *P. oxalicum* 114-2. The PCR products were sequenced to verify the proper fusion of YFP fragments with the target proteins.

**Additional file 5: Figure S3.** The predicted protein–protein docking between PoCreA and PoTup1–Cyc8 complex. (A) The predicted PoTup1/Cyc8 docking model and predicted protein model of PoCreA, respectively. (B) The predicted PoCreA-Tup1/Cyc8 docking model. (C) The predicted PoCreA-Tup1/Cyc8 docking model was rotated 90° clockwise vertically. (D) The predicted PoCreA-Tup1/Cyc8 docking model was rotated 90° clockwise vertically twice.

**Additional file 6: Figure S4.** The original images of Western blot. (A) The anti-H3K4me1 antibody, anti-H3K4me2 antibody, and anti-H3K4me3 antibody were used to detect H3K4 methylation. (B) The anti-H3K36me1 antibody, anti-H3K36me2 antibody, and anti-H3K36me3 antibody were used to detect H3K36 methylation. (C) The anti-H3K79me1 antibody and anti-H3K79me2 antibody were used to detect H3K79 methylation. (D) Equal amounts of the total protein and the anti-histone H3 antibody were set as the loading control.

**Additional file 7: Figure S5. **Domain architecture analysis of Tup1 and Cyc8 in *S. cerevisiae*, *T. reesei* and *P. oxalicum*. (A) Domain architecture analysis of Cyc8 orthologs. (B) Domain architecture analysis of Tup1 orthologs. The SMART server (http://smart.embl-heidelberg.de/) was used for the domain architecture analysis of Tup1p and Cyc8p in *S. cerevisiae*, *P*.* oxalicum*, and *T. reesei*.

**Additional file 8: Figure S6.** The effects of CreA/Cre1 on the expression of *set1*, *set2*, *tup1*, and *cyc8 *in *T. reesei* and* M. grisea* according to their transcriptome data. The transcription data were retrieved from Gene Expression Omnibus (https://www.ncbi.nlm.nih.gov/geo/). The datasets for *T. reesei *Tr*cre1 *deletion strain are GSE57374. The datasets for *M. grisea* Mg*cre1 *deletion strain are GSE153084.

**Additional file 9: Table S2.** Primers used in this study.

## Data Availability

The information about the proteins of *T. reese*i on Table 1 are retrieved from the reference sequence (RefSeq) genome of *T. reesei* QM6a (Accession: PRJNA225530) (https://www.ncbi.nlm.nih.gov/bioproject/PRJNA225530). The information about the proteins of *P. oxalicum* on Table 2 and Table 3 are retrieved from the Whole Genome Shotgun project of *P. oxalicum* (Accession: AGIH00000000.1) (https://www.ncbi.nlm.nih.gov/nuccore/AGIH00000000.1). The information about the proteins of *S. cerevisiae* on Tables 1, 2, and 3 are retrieved from *Saccharomyces* Genome Database (www.yeastgenome.org). All other data that support the findings of this study can be found in Additional files 1, 2, 3, 4, 5, 6, 7, 8 and 9.
