# Supplementary material for: Carbon catabolite repression involves physical interaction of the transcription factor CRE1/CreA and the Tup1–Cyc8 complex in Penicillium oxalicum and Trichoderma reesei
Source: Biotechnol Biofuels. 2021 Dec 24;14:244. doi: 10.1186/s13068-021-02092-9 (PMC8710005; doi:10.1186/s13068-021-02092-9)
Supplement: Supplementary file 1 — Additional file 1: Table S1. The strains used in this study. [file 13068_2021_2092_MOESM1_ESM.docx]

**Table S1** The strains used in this study

| **Strains** | **Genotype** | **Description** | **Reference** |
| --- | --- | --- | --- |
| 114-2 | Wild type | The wild type of *P*. *oxalicum* | [8] |
| QP4 | ΔTr*pyr4*::*hph* | The strain of substituting gene Tr*pyr4* with *hph* marker in *T. reesei* QM9414 | [31] |
| TrCre1-TAP | Tr*cre1*::TAP::*pyrG* | Fusing with the FLAG-HA tag at the C-terminus of TrCre1 | This study |
| PoCreA-TAP | Po*creA*::TAP::*hygA* | Fusing with the FLAG-HA tag at the C-terminus of PoCreA | This study |
| PoCyc8-TAP | Po*cyc8*::TAP::*hygA* | Fusing with the FLAG-HA tag at the C-terminus of PoCyc8 | This study |
| ΔPo*CreA* | ΔPo*creA*::*hph* | The strain of substituting gene Po*creA* with *hph* marker, Po*creA* deletion strain | [8] |
| OEPo*CreA* | *ptrA*::*PgpdA*:: Po*creA* | The strain of Po*creA* overexpression under the promoter of *gpdA* | [16] |
| ΔPo*set2* | ΔPo*set2*::*hph* | The strain of substituting gene Po*set2* with *hph* marker, Po*set2* deletion strain | [40] |
| PoCyc8-YFP-PoCreA | P*PDE_01335*::Po*cyc8*::NYFP::Ter1, *ptrA*; P*gpdA*:: Po*creA*::CYFP::Ter2, *hygA* | The co-expression strain of PoCyc8 fused with N-YFP and PoCreA fused with C-YFP | This study |
| PoTup1-YFP-PoCreA | P*PDE_01335*::Po*tup1*::NYFP::Ter1, *ptrA*; P*gpdA*:: Po*creA*::CYFP::Ter2, *hygA* | The co-expression strain of PoTup1 fused with N-YFP and PoCreA fused with C-YFP | This study |
| PoCyc8-YFP-PoSet2 | P*PDE_01335*::Po*cyc8*::NYFP::Ter1, *ptrA*; P*gpdA*:: Po*set2*::CYFP::Ter2, *hygA* | The co-expression strain of PoCyc8 fused with N-YFP and PoSet2 fused with C-YFP | This study |
| PoCyc8-YFP-empty | P*PDE_01335*::Po*cyc8*::NYFP::Ter1, *ptrA*; P*gpdA*:: CYFP::TER2, *hygA* | The control strain expressing PoCyc8 fused with N-YFP and empty C-YFP | This study |
| PoTup1-YFP-empty | P*PDE_01335*::Po*tup1*::NYFP::Ter1, *ptrA*; P*gpdA*:: CYFP::Ter2, *hygA* | The control strain expressing PoTup1 fused with N-YFP and empty C-YFP | This study |
| empty-YFP-empty | P*PDE_01335*:: NYFP::Ter1, *ptrA*; P*gpdA*:: CYFP::Ter2, *hygA* | The control strain expressing empty N-YFP and empty C-YFP | This study |
