# Supplementary figures and images for: Carbon catabolite repression involves physical interaction of the transcription factor CRE1/CreA and the Tup1–Cyc8 complex in Penicillium oxalicum and Trichoderma reesei
Source: Biotechnol Biofuels. 2021 Dec 24;14:244. doi: 10.1186/s13068-021-02092-9 (PMC8710005; doi:10.1186/s13068-021-02092-9)

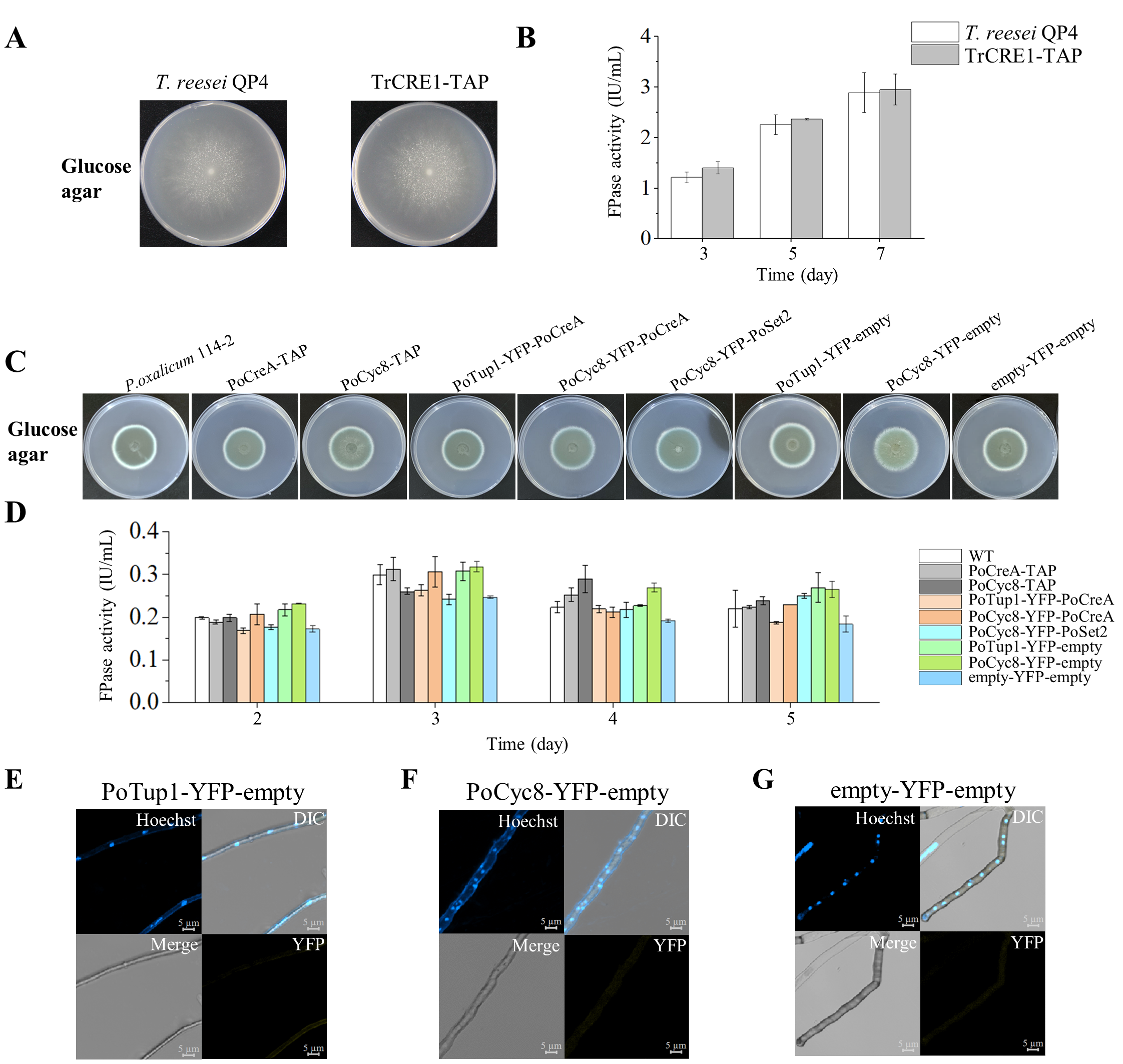

Supplement: Supplementary file 2 — Additional file 2: Figure S1. Phenotypic analysis and enzyme activity determination of TAP and BiFC strains. (A) Growth phenotype of TrCRE1-TAP strain and parent T. reesei QP4. (B) FPA activities assay of TrCRE1-TAP strain and parent T. reesei QP4. (C) Phenotypic analysis of TAP and BiFC strains in P. oxalicum. (D) FPA activities assay of TAP and BiFC strains in P. oxalicum. (E) Microscopy of PoTup1-YFP-empty BiFC strain. (F) Microscopy of PoCyc8-YFP-empty BiFC strain. (G) Microscopy of empty-YFP-empty BiFC strain. [file 13068_2021_2092_MOESM2_ESM.tif]

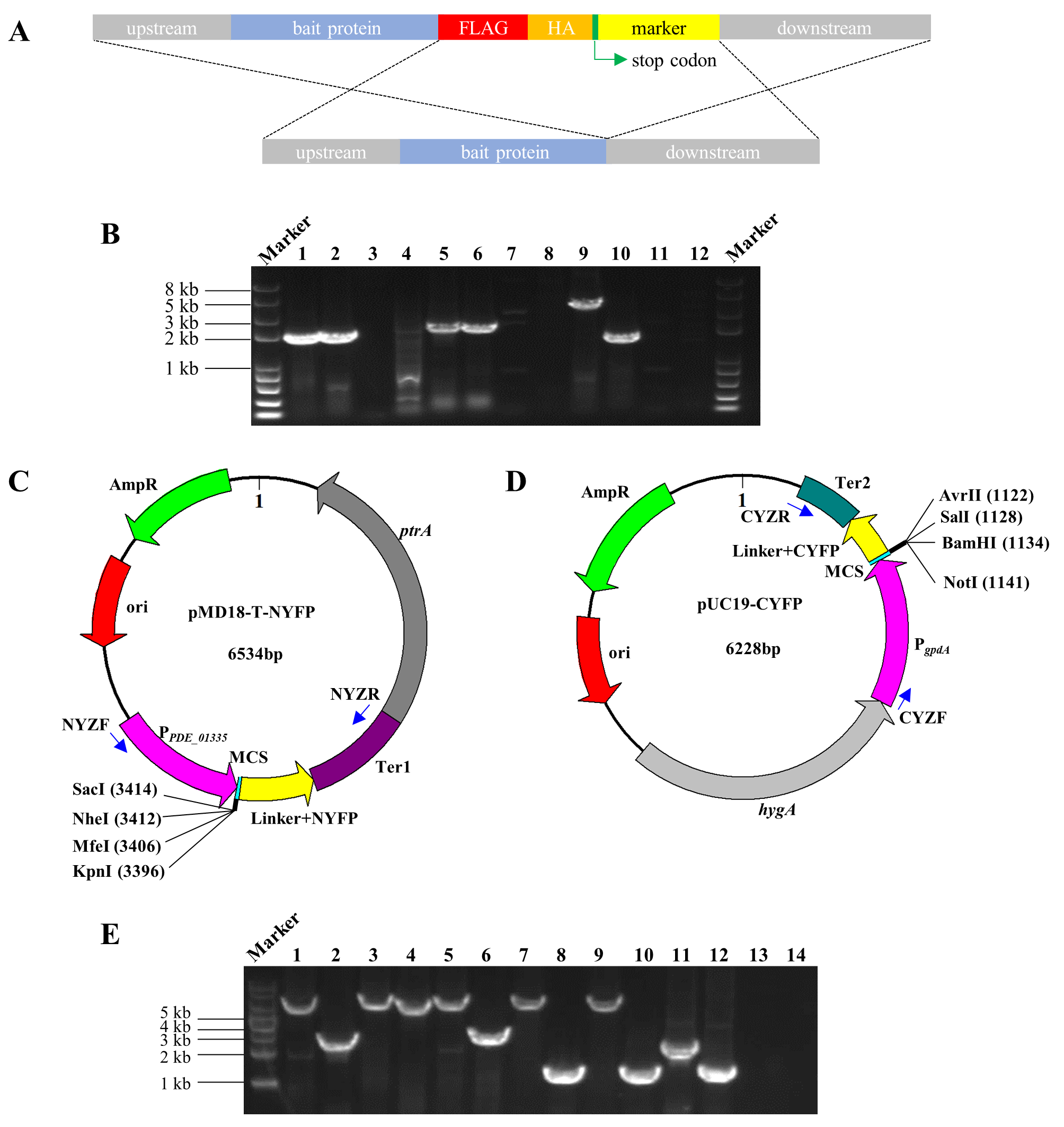

Supplement: Supplementary file 4 — Additional file 4: Figure S2. Construction strategy and verification of TAP and BiFC strains. (A) Construction strategy of TAP strains. (B) Results of diagnostic PCR of TAP strains. Lane 1 (1895 bp) and Lane 2 (1928 bp) represent TrCRE1-TAP (amplified using primers TrCRE1-F/pyrG-YZR and pyrG-YZF/TrCRE1-DR, respectively); lane 3 and Lane 4 represent negative control (T. reesei QP4); lane 5 (2289 bp) and Lane 6 (2234 bp) represent PoCreA-TAP (amplified using primers PoCreA-F/hygA-YZR and hygA-YZF/PoCreA-DR respectively); lane 7 and lane 8 represent negative control (P. oxalicum 114-2); lane 9 (3646 bp) and lane 10 represent PoCyc8-TAP (amplified using primers PoCyc8-F/hygA-YZR and hygA-YZF/PoCyc8-DR respectively); lane 11 and lane 12 represent negative control (P. oxalicum 114-2). The PCR products were sequenced to verify the proper insertion of FLAG-HA tags. (C) Map of pMD18-T-NYFP which carries the N-terminal (1–155 aa) of the YFP. (D) Map of pUC19-NYFP which carries C-terminal (156–238 aa) of the YFP. (E) Results of diagnostic PCR of BiFC strains using primers NYZF/NYZR (lane 1, 3, 5, 7, 9, 11, 13) and CYZF/CYZR (lane 2, 4, 6, 8, 10, 12, 14). Lane 1 (5200 bp) and lane 2 (2740 bp) represent PoCyc8-YFP-PoCreA; lane 3 (5200 bp) and lane 4 (4789 bp) represent PoCyc8-YFP-PoSet2; lane 5 (5217 bp) and lane 6 (2740 bp) represent PoTup1-YFP-PoCreA; lane 7 (5200 bp) and lane 8 (1515 bp) represent PoCyc8-YFP-empty; lane 9 (5217 bp) and lane 10 (1515 bp) represent PoTup1-YFP-empty; lane 11 (2271 bp) and lane 12 (1515 bp) represent empty-YFP-empty; lane 13 and 14 were negative control amplified by template of the parent strain P. oxalicum 114-2. The PCR products were sequenced to verify the proper fusion of YFP fragments with the target proteins. [file 13068_2021_2092_MOESM4_ESM.tif]

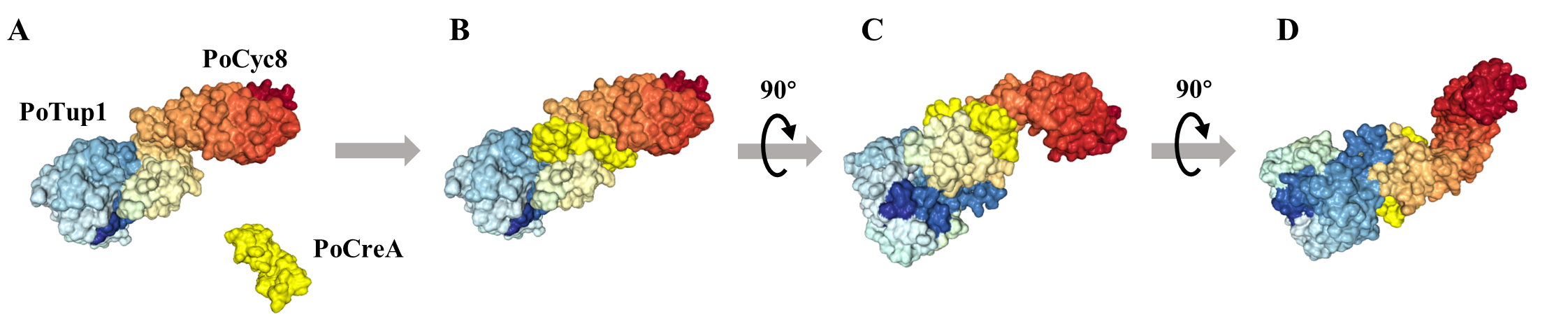

Supplement: Supplementary file 5 — Additional file 5: Figure S3. The predicted protein–protein docking between PoCreA and PoTup1–Cyc8 complex. (A) The predicted PoTup1/Cyc8 docking model and predicted protein model of PoCreA, respectively. (B) The predicted PoCreA-Tup1/Cyc8 docking model. (C) The predicted PoCreA-Tup1/Cyc8 docking model was rotated 90° clockwise vertically. (D) The predicted PoCreA-Tup1/Cyc8 docking model was rotated 90° clockwise vertically twice. [file 13068_2021_2092_MOESM5_ESM.tif]

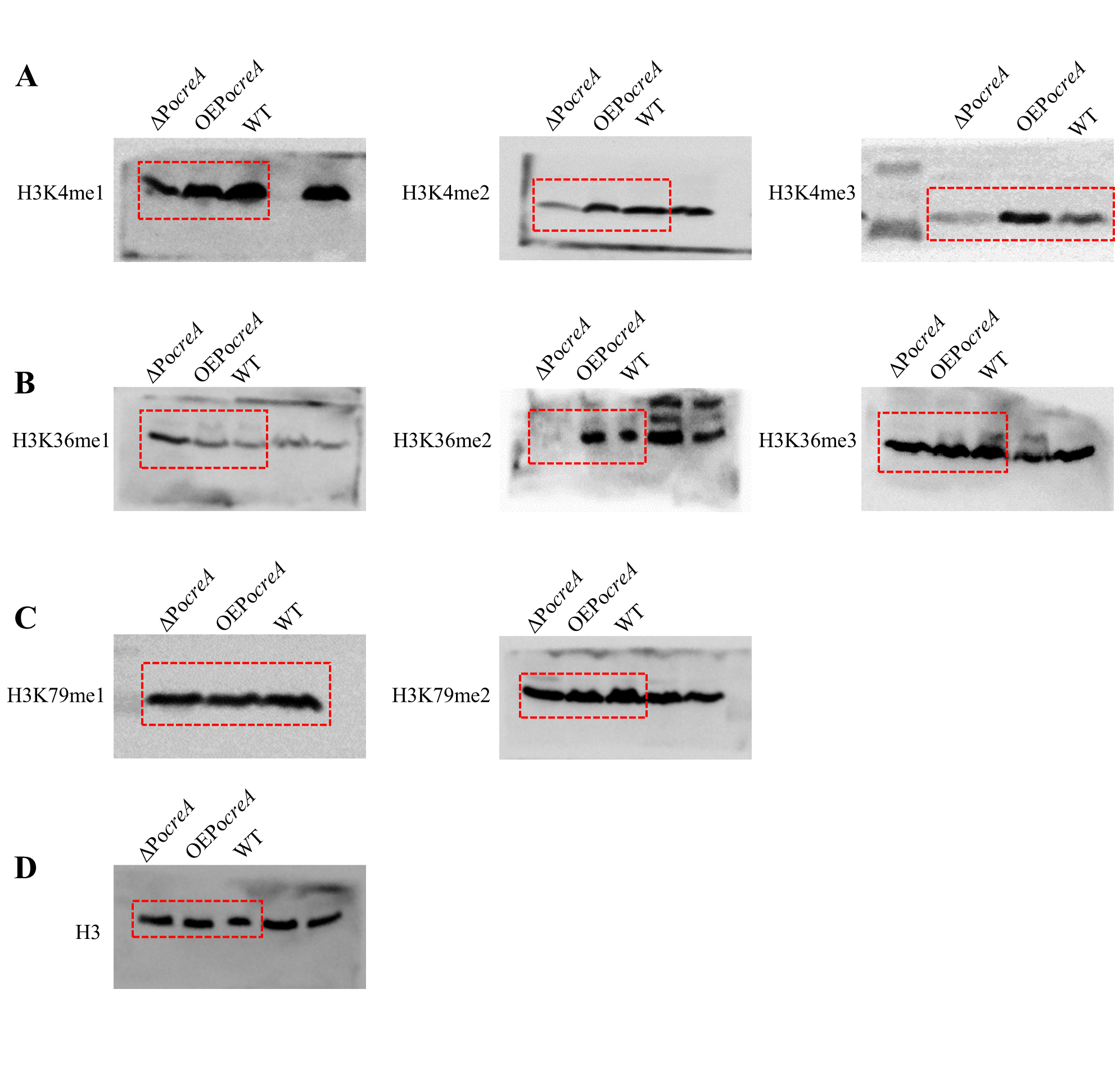

Supplement: Supplementary file 6 — Additional file 6: Figure S4. The original images of Western blot. (A) The anti-H3K4me1 antibody, anti-H3K4me2 antibody, and anti-H3K4me3 antibody were used to detect H3K4 methylation. (B) The anti-H3K36me1 antibody, anti-H3K36me2 antibody, and anti-H3K36me3 antibody were used to detect H3K36 methylation. (C) The anti-H3K79me1 antibody and anti-H3K79me2 antibody were used to detect H3K79 methylation. (D) Equal amounts of the total protein and the anti-histone H3 antibody were set as the loading control. [file 13068_2021_2092_MOESM6_ESM.tif]

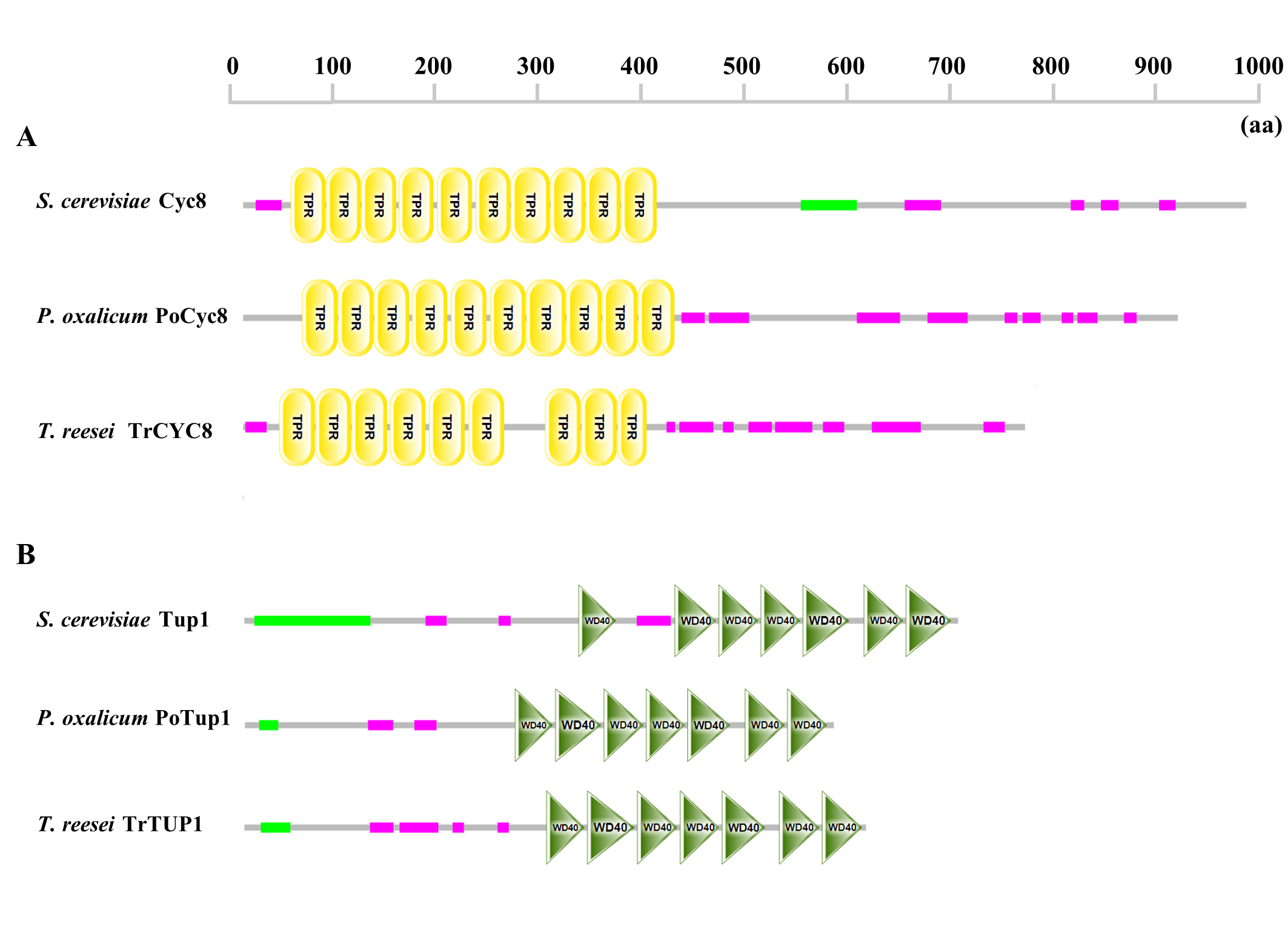

Supplement: Supplementary file 7 — Additional file 7: Figure S5. Domain architecture analysis of Tup1 and Cyc8 in S. cerevisiae, T. reesei and P. oxalicum. (A) Domain architecture analysis of Cyc8 orthologs. (B) Domain architecture analysis of Tup1 orthologs. The SMART server (http://smart.embl-heidelberg.de/) was used for the domain architecture analysis of Tup1p and Cyc8p in S. cerevisiae, P. oxalicum, and T. reesei. [file 13068_2021_2092_MOESM7_ESM.tif]

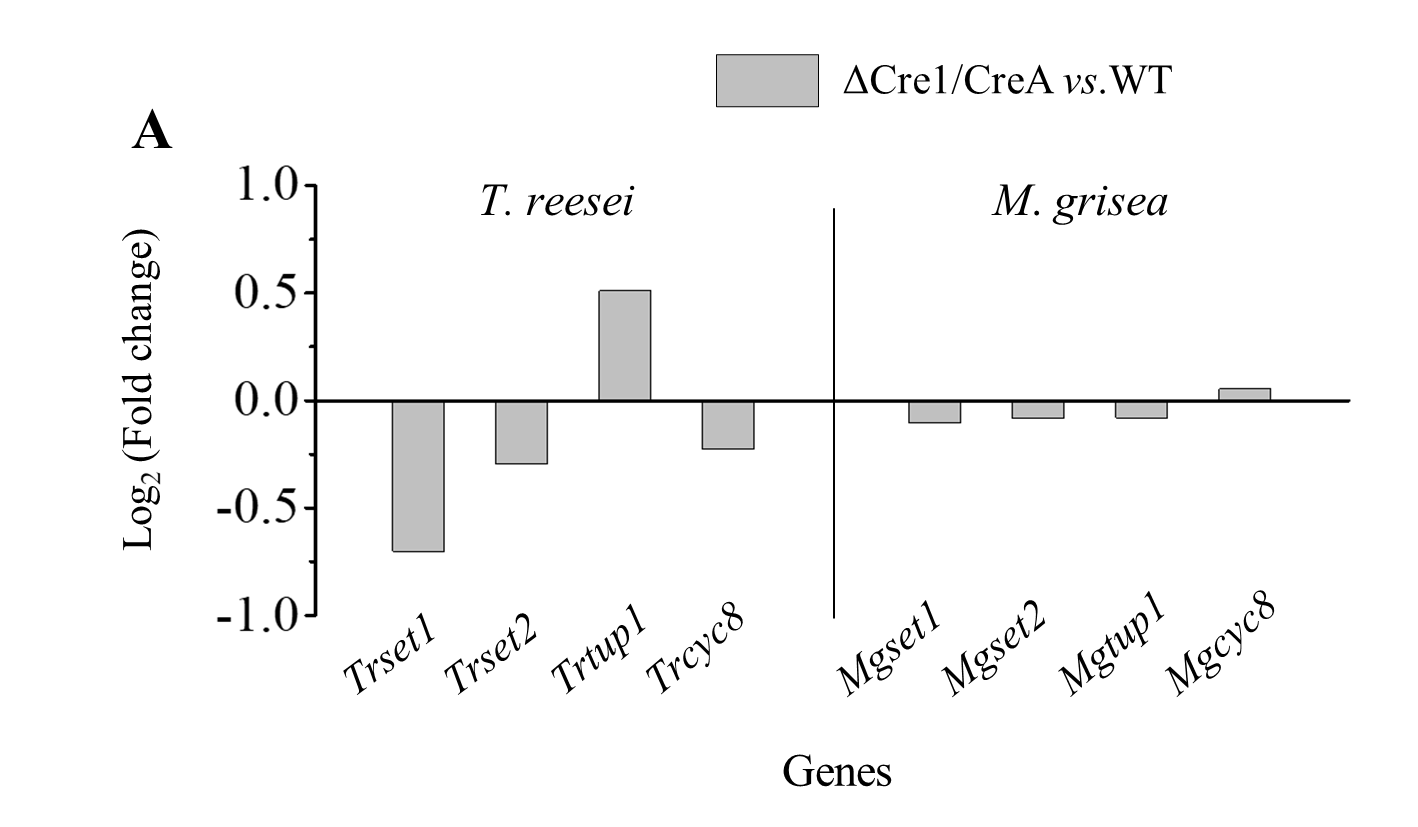

Supplement: Supplementary file 8 — Additional file 8: Figure S6. The effects of CreA/Cre1 on the expression of set1, set2, tup1, and cyc8 in T. reesei and M. grisea according to their transcriptome data. The transcription data were retrieved from Gene Expression Omnibus (https://www.ncbi.nlm.nih.gov/geo/). The datasets for T. reesei Trcre1 deletion strain are GSE57374. The datasets for M. grisea Mgcre1 deletion strain are GSE153084. [file 13068_2021_2092_MOESM8_ESM.tif]
