# Supplementary material for: Carbon catabolite repression involves physical interaction of the transcription factor CRE1/CreA and the Tup1–Cyc8 complex in Penicillium oxalicum and Trichoderma reesei
Source: Biotechnol Biofuels. 2021 Dec 24;14:244. doi: 10.1186/s13068-021-02092-9 (PMC8710005; doi:10.1186/s13068-021-02092-9)
Supplement: Supplementary file 9 — Additional file 9: Table S2. Primers used in this study. [file 13068_2021_2092_MOESM9_ESM.docx]

**Table S2** Primers used in this study

| **Primers** | **Primer sequence** |
| --- | --- |
| TrCRE1-F | CGTTCTCCCTCGATGAACGAAG |
| TrCRE1-*tap*-R | GGCGTAGTCGGGGACGTCGTAGGGGTACTTGTCGTCGTCGTCCTTGTAGTCCATCCGATCCATGAGGTCGCCACC |
| TrCRE1*-*DF | GTGTAACGGTATTGACTAAAAGGGAATGTCCGGTACTCATGGCGCG |
| TrCRE1*-*DR | CAGACATCATCATCGGGATCAGC |
| *pyrG-*F | GACAAGTACCCCTACGACGTCCCCGACTACGCCTAGGCAACTTCCTCGAGAACGCGC |
| *pyrG-*R | CCCTTTTAGTCAATACCGTTACAC |
| TrCRE1-CSF | CCACACTCACTCACACACCCTC |
| TrCRE1-CSR | GAAGATGGTTGGGCTTCGCTCACC |
| *pyrG*-YZF | GAGTGAGGTGCTGCCCGAACAG |
| *pyrG*-YZR | CGACTATCATTCAAACAGACCAC |
| PoCreA-F | CCTTGTCACGATCCCGATAAGTC |
| PoCreA-*tap*-R | TTAGGCGTAGTCGGGGACGTCGTAGGGGTACTTGTCGTCGTCGTCCTTGTAGTCAAAGCGATCGGCCAGATCATTG |
| PoCyc8F | TGGACCTCTTCACTTCCATCCTC |
| PoCyc8-*tap*-R | TTAGGCGTAGTCGGGGACGTCGTAGGGGTACTTGTCGTCGTCGTCCTTGTAGTCGGCAGTCTCAGTCTTG |
| PoCreA*-*DF | CCGTCACCAGCCCCTGGGTTGAAAGAAAAGCCTTCGACTC |
| PoCreA*-*DR | CCTTCAGGACCGTACTAAACAC |
| PoCyc8-DF | CCGTCACCAGCCCCTGGGTTGAGTTGACAAAAGTACGCATTG |
| PoCyc8-DR | CCTCCTATTAGCTTCGTGTAGTC |
| *hygA*-F | GACTACAAGGACGACGACGACAAGTACCCCTACGACGTCCCCGACTACGCCTAACGACGTTAACTGATATTG |
| *hygA*-R | CAACCCAGGGGCTGGTGACGG |
| PoCreA-CSF | CCCACCCCGATTTCTCCCTTCC |
| PoCreA-CSR | GATAGTGCAGTTAGTCAAGTAAG |
| PoCyc8-CSF | ACGCGCCCGTCTCCACCAACAAG |
| PoCyc8-CSR | CAGAATACTAGCAGTCTGCACGAGT |
| hygA-YZF | CATTAATGCATTGGACCTCGCAAC |
| hygA-YZR | GCCGCCGCTACTGCTACAAGTGG |
| qPo*cbh1*F | CCACCACCACTACCAGCAAGG |
| qPo*cbh1*R | GTAGCCAACACCACCGCACT |
| qPo*egl1*F | ACCGCTGCTCAGACCACGAC |
| qPo*egl1*R | TGGGTCCCGAGTAGCCAACG |
| qPo*set1*F | TGCCGACATCAATGCTCAGAAG |
| qPo*set1*R | TTCTCCTCGGCGTATAGTCCC |
| qPo*set2*F | GCGAAAAGCATCAAAAAACGG |
| qPo*set2*R | TTTGACTCTGTCGCCGAAGCC |
| qPo*tup1*F | GTTCACTTGACAAGAGTGTACGTGTC |
| qPo*tup1*R | GTCTTGTCAAGACTGCCACTGACCAGG |
| qPo*cyc8*F | CTATCAAGAAACAGGCGAGCGATGAG |
| qPo*cyc8*R | CTCAGAGGAACTACGTCGCTGCATCTG |
| qPo*actin*F | GTTCCATTCTCGCCTCCCTCT |
| qPo*actin*R | AGAAGCACTTGCGGTGAACGA |
| Po*cbh1*-1F | CATATCCTTCACCGAGTGGGTTG |
| Po*cbh1*-1R | GACGATGGAGGCGAAATTGAGG |
| Po*cbh1*-2F | CTGGGGAGCTTCCTCCGTGTCCG |
| Po*cbh1*-2R | CAATTCGCCTCTCGACTGATTTC |
| Po*cbh1*-3F | CTTCAGGATCCACCCAAGGAAG |
| Po*cbh1*-3R | GTGATGGATTGGATCAAAGATC |
| Po*egl1*-1F | CAGATGCATCATCTCTCACCTG |
| Po*egl1*-1R | CCTTGGGGTGAAACTCTTCAAC |
| Po*egl1*-2F | GCGTCTCCGGATCGTTTCGCTATG |
| Po*egl1*-2R | CTGGTCAAGTCTCCGTGTTCCTTC |
| Po*egl1*-3F | GTCGAAATACGACAGAAATGATG |
| Po*egl1*-3R | GTTGAGCAAACTTGGCAATGAGC |
| PoTup1-NF | CAAATCCAACCTTCACCACCTCATGTACAATACGCATCGCGGC |
| PoTup1-NR | GGAGCGGCGGCCGCTGTGCCGTCCTGTGTATGTCGAGTACCTG |
| PoCyc8-NF | CAAATCCAACCTTCACCACCTCATGTCGCACACCCAACCATCTCCC |
| PoCyc8-NR | GATGGAGCGGCGGCCGCTGTGGGCAGTCTCAGTCTTGGAAGGAG |
| PoCreA-CF | CATTCTTACGACTGCCGTCAATGTCTTCTTCAACCTCAGTGG |
| PoCreA-CR | GGGATCTTGCAGGCCGGGCGAAAGCGATCGGCCAGATCATTGC |
| PoSet2-CF | CATTCTTACGACTGCCGTCAATGTCCGCTCATGGCAACGCGG |
| PoSet2-CR | CGGGATCTTGCAGGCCGGGCGTGATTGCCCATGTACACCCACCG |
| NYZF | CAATCCCGACTCATTACATACCTCCA |
| NYZR | GAGATAGAGAATTGTGTGGGATGAG |
| CYZF | GTCAAGAGACCTACGAGACTGAG |
| CYZR | CGTGAACAAGGGACATTACAATG |
